# Tissue distribution of *Histoplasma capsulatum* in deceased people living with HIV assessed by minimally invasive tissue sampling: An autopsy-based case series in the Brazilian Amazon

**DOI:** 10.1371/journal.pntd.0014545

**Published:** 2026-08-06

**Authors:** Karolina Jeaneth Solórzano-Chavarría, Taygo Silva Graciliano, Izabella Picinin Safe Lacerda, Alexandre Vilhena Silva-Neto, Luiz Ferreira, Monique Freire Santana, Katia Santana Cruz, Carla Silvana Silva Santos, Juan Diego Ribeiro Almeida, Luciana Aires Oliveira, Jaume Ordi, Natalia Rakislova, Mikel Martinez, Juan Carlos Hurtado, João Vicente Braga Souza, Quique Bassat, Marcus Vinícius Guimarães Lacerda, Djane Clarys Baía-da-Silva

**Affiliations:** 1 Fundação de Medicina Tropical Dr. Heitor Vieira Dourado, Manaus, Brazil; 2 Universidade do Estado do Amazonas, Manaus, Brazil; 3 Universidade Federal do Amazonas, Hospital Universitário Getúlio Vargas, Manaus, Brazil; 4 Instituto Nacional de Pesquisas da Amazônia, Manaus, Brazil; 5 Pathology department of the Hospital Clínic of Barcelona, Barcelona, Spain; 6 Facultat de Medicina i Ciències de la Salut, Universitat de Barcelona (UB), Barcelona, Spain; 7 CIBER de Epidemiología y Salud Pública, Instituto de Salud Carlos III, Madrid, Spain; 8 Microbiology Department, Hospital Clínic-Universitat de Barcelona, Barcelona, Spain; 9 Barcelona Institute for Global Health (ISGlobal), Barcelona, Spain; 10 Centro de Investigación Biomédica en Red de Enfermedades Infecciosas (CIBERINFEC), Instituto de Salud Carlos III, Madrid, Spain; 11 Centro de Investigação em Saúde de Manhiça (CISM), Maputo, Mozambique; 12 ICREA, Pg. Lluís Companys, Barcelona, Spain; 13 Institut Clínic de Medicina I Dermatologia, Hospital Clínic de Barcelona, Barcelona, Spain; 14 Pediatrics Department, Hospital Sant Joan de Déu, Universitat de Barcelona, Esplugues, Barcelona, Spain; 15 CIBER de Epidemiología y Salud Pública, Instituto de Salud Carlos III, Madrid, Spain; 16 Instituto Leônidas & Maria Deane, Fiocruz Amazônia, Manaus, Brazil; 17 Universidade Nilton Lins, Manaus, Brazil; FIOCRUZ: Fundacao Oswaldo Cruz, BRAZIL

## Abstract

This observational autopsy-based case series characterized the tissue distribution of *Histoplasma capsulatum* in deceased people living with HIV (PLHIV) using minimally invasive tissue sampling (MITS). This study included 59 deceased PLHIV admitted to a tertiary referral hospital in the Brazilian Amazon between October 2020 and June 2022. Tissue and body fluid samples were analyzed using fungal culture, histopathology, conventional polymerase chain reaction (PCR), and quantitative PCR (qPCR). *H. capsulatum* was detected in 27 of 59 (45.8%) patients, including nine (33.3%) with multi-organ detection of *H. capsulatum*. The liver (9/27, 33.3%) and lungs (8/27, 29.6%) were the most frequently involved organs in this series. qPCR amplification was detected across multiple tissues, with lower median Ct values in the lung (40.3, range 37.0–45.0) and liver (41.0, range 38.0–44.0) than in cerebrospinal fluid (43.5, range 40.0–48.0) and brain tissue (44.0, range 41.0–47.0), suggesting greater fungal DNA detection in the lungs and liver. Molecular methods detected fungal DNA in tissue samples that were negative by culture, although several amplification signals occurred near the assay detection limit. *H. capsulatum* showed heterogeneous tissue distribution in deceased PLHIV, with the liver and lungs being the most frequently involved organs. Molecular methods complemented conventional diagnostic techniques by identifying fungal DNA in culture-negative tissues, providing a more comprehensive assessment of tissue involvement. These findings improve understanding of disseminated histoplasmosis in advanced HIV infection and may inform future tissue-based diagnostic strategies.

## Author summary

Histoplasmosis is a serious fungal infection that causes significant morbidity and mortality in people living with the human immunodeficiency virus, especially in endemic regions. Diagnosis remains challenging because its symptoms resemble those of other opportunistic infections, and sensitive laboratory tests are often unavailable. In this study, we analyzed postmortem tissue samples collected via a minimally invasive procedure to investigate the distribution of the fungus throughout the body. Almost half of the patients presented with histoplasmosis, with the lungs and liver being the most frequently affected organs. Molecular methods detected fungal DNA in additional tissue samples that were negative by fungal culture, particularly in samples with low fungal DNA burden. Although based on postmortem samples, our findings contribute to understanding disease dissemination and reinforce the complementary role of molecular techniques in diagnosis in endemic areas.

## Introduction

Histoplasmosis, an AIDS-defining opportunistic infection, is a systemic fungal infection primarily caused by *Histoplasma capsulatum* var. *capsulatum*, with *H. capsulatum* var. *duboisii* occurring mainly in African settings [1]. The fungus thrives in soil enriched with bird or bat droppings, which explains its broad geographic distribution across endemic regions, including Latin America (e.g., Brazil, Colombia, Venezuela, and Guatemala) and parts of Africa and Asia [2–5]. Despite this distribution, histoplasmosis remains underrecognized in many endemic settings, and underdiagnosis may contribute to increased morbidity and mortality, particularly among people living with HIV (PLHIV), in whom the disease often presents in disseminated forms [4,6,7].

One of the main challenges in the clinical management of histoplasmosis is its frequent misdiagnosis as tuberculosis, given the substantial overlap in clinical and radiological characteristics [5,8]. This diagnostic difficulty is further compounded by limited access to specific diagnostic tools in many endemic settings [9]. Although urine or blood antigen detection assays for *Histoplasma* offer high sensitivity, especially in disseminated disease, these methods are rarely available within public health systems in many endemic countries [10,11].

In immunocompetent individuals, histoplasmosis is often asymptomatic or self-limiting. In contrast, in immunosuppressed individuals, especially PLHIV, the infection frequently disseminates, affecting multiple organs and leading to severe clinical outcomes [12,13]. Disseminated histoplasmosis (DH) is associated with rapid clinical deterioration and high mortality rates when diagnosis and treatment are delayed [14,15]. Accurate diagnosis requires a combination of histopathology, culture, and molecular methods, which often require invasive procedures to obtain tissue samples [16].

These diagnostic challenges highlight the importance of understanding the distribution of *H. capsulatum* across different organs. This is particularly relevant in resource-limited settings, where access to diagnostic tools is often restricted. However, evidence remains limited to which tissues are more likely to yield detectable *H. capsulatum* DNA, particularly with minimally invasive postmortem approaches.

Minimally Invasive Tissue Sampling (MITS) is a postmortem technique for collecting tissue samples from key organs [17,18]. It allows sampling without the need for a full autopsy or imaging guidance. This method has emerged as a valuable tool for determining the cause of death in infectious diseases [19]. It is particularly useful in low-resource and culturally sensitive settings due to its feasibility and acceptability [19,20]. In addition, MITS provides an opportunity to systematically investigate tissue involvement in disseminated infections [19,21].

To fill this knowledge gap, we conducted a systematic post-mortem investigation describing the tissue distribution of *H. capsulatum* in deceased HIV-positive individuals using MITS. Unlike previous studies that focused primarily on clinical presentation or diagnostic performance evaluation, our work integrates fungal culture, histopathology, conventional PCR, and quantitative PCR to provide a comprehensive characterization of fungal dissemination across multiple organs. By combining complementary diagnostic methods within a standardized MITS protocol, this research generates new pathological evidence on disseminated histoplasmosis in the Brazilian Amazon, establishing a solid foundation for future tissue-based diagnostic and mortality surveillance studies in endemic regions.

## Methods

### Ethical aspects

This study was approved by the Research Ethics Committee of Fundação de Medicina Tropical Dr. Heitor Vieira Dourado (FMT-HVD), Brazil (CAAE: 34846620.8.0000.0005). All procedures were conducted in accordance with national and international ethical standards, including the Declaration of Helsinki and applicable Brazilian regulations. Written informed consent for the MIT sampling procedure was obtained from each deceased participant’s next of kin prior to postmortem sample collection, in accordance with institutional protocols. All data were anonymized prior to analysis to ensure participant confidentiality.

### Study setting

This is an observational autopsy-based case series study conducted at FMT-HVD, a tertiary referral hospital specializing in the diagnosis and treatment of tropical diseases, including fungal infections [22]. The hospital is in Manaus, in the state of Amazonas, in Brazil’s Northern Region. The city has an estimated population of 2 million, with the majority residing in urban and peri-urban areas [23]. A previous study conducted at this hospital showed a high incidence of histoplasmosis (34%) among autopsied patients [14]. This substantial burden highlights the need to better characterize the tissue distribution of *H. capsulatum* in this setting.

### Patients included

Patients who died at the FMT-HVD between October 2020 and June 2022 were included if they met the following criteria: (1) age ≥ 18 years with a confirmed diagnosis of HIV; (2) written informed consent for MITS provided by relatives; and (3) a postmortem interval of less than 48 hours. Cases with a postmortem interval exceeding 48 hours or with insufficient tissue quality due to advanced autolysis were excluded. No exclusions were made based on the presence of coinfections, as these were considered part of the clinical spectrum of advanced HIV infection. HIV infection was confirmed based on documented medical records and laboratory diagnostic criteria in accordance with national guidelines [24].

### Demographic, epidemiological, and clinical information

Demographic, epidemiological, and clinical information were extracted from electronic medical records using a standardized data collection form. Variables collected included age, sex, HIV-related parameters (CD4 ⁺ cell count, viral load, and antiretroviral therapy use), presence of opportunistic infections, prior or inpatient antifungal treatment, comorbidities, and selected laboratory parameters (e.g., hematological and biochemical profiles), when available. Clinical data were obtained at the time of HIV diagnosis, before hospitalization, and during the hospital stay to capture each patient’s clinical trajectory.

Epidemiological data included place of residence (e.g., urban or peri-urban areas) and, when available, geographic origin. Information on occupational exposure, travel history, and specific environmental factors was not systematically recorded in the medical records, preventing a consistent analysis of these aspects.

Data extraction was performed by trained researchers following a standardized protocol. A subset of the cases was independently reviewed to ensure consistency of information, and any discrepancies were resolved by consensus or, when necessary, by consulting a third reviewer. Missing data were recorded as unavailable and were not imputed.

### Minimally invasive tissue sampling procedure

Participants underwent tissue collection using MITS, as previously described [20,25]. The protocol included a detailed external macroscopic examination followed by percutaneous sampling using a 14-gauge needle. Samples were collected from the lungs, liver, brain, ileum, blood, and cerebrospinal fluid (CSF), as well as pleural and ascitic fluid when present.

Sampling was performed using a standardized protocol, with needle insertions guided by anatomical landmarks specific to each organ. Whenever feasible, 2–4 punctures per organ were performed, with sampling depth adjusted to reach the target parenchyma. Tissue fragments of approximately 25–50 mg were obtained for solid samples, while liquid samples had a minimum volume of approximately 200 µL. To minimize cross-contamination, a new sterile needle was used for each organ, and strict aseptic technique was maintained throughout the procedure.

Each sample was assigned a unique study code and, when sufficient material was available, divided into three analytical pipelines:

(i) mycological analysis (Mycology Laboratory, FMT-HVD);
(ii) molecular analysis (stored in DNase-free tubes and transported under cold chain at 2–8 °C);
(iii) histopathological analysis (fixed in 10% neutral buffered formalin).

Although multiple samples per organ were obtained whenever possible, the amount of tissue collected may have been limited by post-mortem factors, including tissue autolysis, anatomical accessibility, and the inherent constraints of the MITS procedure, which prioritizes minimal invasiveness while maintaining adequate tissue representativeness. When sample volume or weight was limited, a predefined prioritization strategy (histopathology → mycology → molecular analysis) was applied and documented for each case.

All samples were coded, recorded on a tracking form, and transported at a controlled temperature (2–8 °C), processed on the same day whenever possible, or stored according to each analytical method’s requirements.

### Mycological analysis

Mycological analyses were performed according to established mycological criteria [26]. Solid tissue samples were fragmented under sterile conditions and inoculated onto culture media, including Sabouraud agar, Mycosel agar, brain–heart infusion (BHI) agar, and Niger seed agar. Approximately 40 µL of the liquid samples were also inoculated onto the same media.

Cultures were incubated at 25–30 °C for filamentous growth and at 37 °C for yeast-phase growth and monitored for 30–45 days. Fungal growth compatible with *H. capsulatum* within this incubation period was considered positive. Repeat cultures were not performed systematically and depended on sample availability.

Identification of *H. capsulatum* was based on classical macroscopic and microscopic morphological characteristics, including microscopic examination using lactophenol cotton blue staining and demonstration of thermal dimorphism through conversion between mycelial and yeast phases, in accordance with standard mycological criteria [27,28]. Molecular detection by PCR was performed in parallel but was not used as a confirmatory method for culture results.

All procedures followed biosafety recommendations for handling dimorphic pathogenic fungi and were conducted in laboratory facilities equipped to safely manipulate *Histoplasma* spp. (biosafety level 3 with enhanced precautions, according to institutional protocols).

### Molecular methods

Conventional PCR and qPCR were performed independently as complementary molecular methods. Molecular detection was defined as a positive result by either conventional PCR or qPCR. Molecular analyses were performed according to the methodology described by Silva et al. [29]. DNA was extracted from clinical samples using the Invitrogen Blood & Tissue Kit from 200 µL of body fluid or 25–50 mg of tissue, and eluates (50 µL) were stored at −80 °C.

A TaqMan-based qPCR assay targeting the ITS1 region of *H. capsulatum* was performed using primers HcITS-106F and HcITS-205R and probe HcITS-127P, as previously described by Buitrago et al. [30], with oligonucleotide conditions optimized by Silva et al. [29]. Reactions were carried out in a final volume of 20 µL containing 1 × TaqMan Master Mix, 300 nM of each primer, and 200 nM probe. Amplification was performed under the following conditions: 95 °C for 10 minutes, followed by 40 cycles of 95 °C for 15 seconds and 60 °C for 60 seconds.

Samples with Ct values ≤38 were considered positive. Amplification signals with Ct values >38 were classified as low-level molecular detections and interpreted with caution, as they occurred near the analytical detection limit and may reflect low fungal DNA burden, residual DNA, or post-mortem degradation. Ct values were interpreted as qualitative or semi-quantitative indicators of fungal DNA detection and should not be considered direct measures of viable fungal burden or clinical disease severity. Ct values were not used as a direct measure of diagnostic accuracy.

All reactions were performed in duplicate and included positive and no-template (negative) controls. An internal β-actin target was included to monitor DNA extraction efficiency and potential PCR inhibition. Samples with absent β-actin amplification or β-actin Ct > 35 were re-tested after 1:5 dilution to assess inhibition. Discordant molecular results between conventional PCR and qPCR may reflect differences in analytical sensitivity, stochastic amplification near the detection limit, DNA degradation in postmortem samples, or variability in the distribution of fungal DNA in tissue [31–34].

Laboratory procedures followed a unidirectional workflow with physically separated areas for DNA extraction, reagent preparation, and amplification to minimize contamination.

The analytical performance of the qPCR assay, including limit of detection, sensitivity, specificity, and amplification efficiency, has been previously described by Silva et al. [29] and was not re-evaluated in this study. Validation in post-mortem samples was made by Silva et al. [29]. Although the assay has demonstrated high analytical specificity, potential cross-reactivity with phylogenetically related fungi cannot be completely excluded.

### Histopathological methods

After fixation in neutral buffered formalin for 24 hours, tissue samples were routinely processed with paraffin embedding and stained using hematoxylin and eosin (H&E) as well as Grocott–Gomori methenamine silver (GMS). When indicated, additional staining with periodic acid–Schiff (PAS) was also performed. Histopathological identification of *H. capsulatum* relied on characteristic morphological features observed in tissue sections, including small yeast forms measuring approximately 2–5 µm in diameter, predominantly intracellular localization within macrophages, and narrow-based budding. Special stains, particularly GMS and PAS, were used to enhance visualization of fungi and facilitate morphological identification. All histopathological assessments were conducted by an experienced pathologist.

### Attribution of the cause of death

After completion of the pathological examination, an infectious disease pathologist reviewed all pathological findings obtained from the MITS procedure. Based on these findings, the contribution of *H. capsulatum* to death was classified as primary, contributory, or unrelated. When necessary, individual cases were discussed with the study investigators to resolve uncertainties and reach a consensus.

### Histoplasmosis classification

Histoplasmosis cases were classified using operational categories developed for this descriptive postmortem study to facilitate characterization of tissue distribution. The predefined patterns of tissue involvement were classified as: (i) multi-organ detection, defined as detection of *H. capsulatum* in two or more organs and/or body fluids; (ii) lung-restricted detection, defined as detection restricted to the lungs; and (iii) single-organ/body fluid detection, defined as detection confined to a single non-pulmonary organ or body fluid. These categories were intended to describe patterns of tissue involvement rather than to establish clinical diagnostic criteria.

### Statistical analysis

Given the case series design, analyses were primarily descriptive and exploratory. Categorical variables were summarized as absolute and relative frequencies [n/N (%)], while continuous variables were described using median and interquartile range (IQR) or mean ± standard deviation, as appropriate. The distribution of *H. capsulatum* detection across tissues was described according to predefined patterns of tissue involvement (multi-organ detection, lung-restricted detection, and single-organ/body-fluid detection). Ct values were interpreted descriptively and compared across tissues to characterize patterns of fungal DNA distribution, without assessing diagnostic accuracy. According to the standard curve, the assay showed reliable amplification up to approximately Ct 37.6. Therefore, amplification detected at Ct values ≥38 was considered close to the technical detection limit and interpreted cautiously as low-level. Missing data were recorded as “not available” and were not imputed. Statistical analyses were performed using R software (version 3.6.0; R Foundation for Statistical Computing, Vienna, Austria).

## Results

### Study population

A total of 59 deceased PLHIV were included in the study. The median age was 40 years (IQR 31–47), and most participants were cisgender men (42/59, 71%). The majority resided in urban areas (55/59, 93%), particularly in the metropolitan region of Manaus (49/59, 83%). Advanced HIV disease was common. Among patients with available data, 20/24 (83%) had CD4 + T-cell counts <200 cells/µL at diagnosis, 13/23 (57%) had HIV viral loads ≥100,000 copies/mL, and 22/46 (48%) were receiving antiretroviral therapy at hospital admission. Respiratory symptoms (42/53, 79%), weight loss (36/52, 69%), and fever (33/53, 62%) were the most frequent clinical manifestations. Severe illness was reflected by high rates of ICU admission (26/55, 47%), invasive mechanical ventilation (29/47, 62%), and vasoactive drug use (30/43, 70%). Opportunistic infections were identified in 20/36 (56%) patients, most commonly tuberculosis and pneumocystosis (Table 1).

**Table 1:** Demographic, clinical, and laboratory characteristics of the study population according to histoplasmosis status.

Characteristic	Overall	Histoplasmosis negative	Histoplasmosis positive
	N = 59	N = 32	N = 27
**Age in years**	40 (31, 47), [59]	40 (31, 47), [32]	40 (33, 47), [27]
**Sex at birth**	17/59 (29%)	12/32 (38%)	5/27 (19%)
**Gender identity**			
Cisgender man	42/59 (71%)	20/32 (63%)	22/27 (81%)
Cisgender woman	17/59 (29%)	12/32 (38%)	5/27 (19%)
**Place of residence**			
Manaus metropolitan area	49/59 (83%)	27/32 (84%)	22/27 (81%)
Municipalities in the interior of Amazonas	10/59 (17%)	5/32 (16%)	5/27 (19%)
**Origin**			
Rural	4/59 (6.8%)	3/32 (9.4%)	1/27 (3.7%)
Urban	55/59 (93%)	29/32 (91%)	26/27 (96%)
**Smoker**	17/46 (37%)	5/28 (18%)	12/18 (67%)
**Alcohol user**	19/45 (42%)	6/27 (22%)	13/18 (72%)
**Recreational drug user**	10/44 (23%)	3/26 (12%)	7/18 (39%)
**Time since HIV infection diagnosis (months)**	14 (1, 83), [37]	57 (1, 95), [21]	3 (1, 53), [16]
**CD4 + T-cell count at HIV diagnosis**			
<200	20/24 (83%)	10/11 (91%)	10/13 (77%)
200-350	3/24 (13%)	0/11 (0%)	3/13 (23%)
350-500	1/24 (4.2%)	1/11 (9.1%)	0/13 (0%)
>=500	0/24 (0%)	0/11 (0%)	0/13 (0%)
**Viral load categories at HIV diagnosis**			
<50	2/23 (8.7%)	2/11 (18%)	0/12 (0%)
50-10000	2/23 (8.7%)	2/11 (18%)	0/12 (0%)
10000-100000	6/23 (26%)	3/11 (27%)	3/12 (25%)
>=100.000	13/23 (57%)	4/11 (36%)	9/12 (75%)
**Weight (in kg) at diagnosis**	60 (48, 66), [24]	60 (49, 72), [14]	58 (42, 60), [10]
**Number of previous hospitalizations**	0.00 (0.00, 0.00), [59]	0.00 (0.00, 1.00), [32]	0.00 (0.00, 0.00), [27]
**On regular ART at admission**	22/46 (48%)	12/27 (44%)	10/19 (53%)
**Concomitant opportunistic diseases at current admission**	20/36 (56%)	9/16 (56%)	11/20 (55%)
Pulmonary tuberculosis	10/20 (50%)	5/9 (56%)	5/11 (45%)
Extrapulmonary tuberculosis	2/20 (10%)	2/9 (22%)	0/11 (0%)
Cutaneous Kaposi’s sarcoma	2/20 (10%)	0/9 (0%)	2/11 (18%)
Visceral Kaposi’s sarcoma	2/20 (10%)	0/9 (0%)	2/11 (18%)
Pneumocystosis	5/20 (25%)	3/9 (33%)	2/11 (18%)
Neurotoxoplasmosis	1/20 (5.0%)	1/9 (11%)	0/11 (0%)
Esophageal candidiasis	3/20 (15%)	3/9 (33%)	0/11 (0%)
Neurocryptococcosis	0/20 (0%)	0/9 (0%)	0/11 (0%)
PML	1/20 (5.0%)	0/9 (0%)	1/11 (9.1%)
**ICU admission**	26/55 (47%)	14/31 (45%)	12/24 (50%)
**ICU length of stay (in days)**	8 (2, 12), [20]	5 (1, 14), [8]	8 (4, 11), [12]
**Need for intubation**	29/47 (62%)	18/27 (67%)	11/20 (55%)
**Need for vasoactive drugs**	30/43 (70%)	18/24 (75%)	12/19 (63%)
**Need for hemodialysis**	5/43 (12%)	3/23 (13%)	2/20 (10%)
**Symptoms at hospitalization**			
Fever	33/53 (62%)	16/27 (59%)	17/26 (65%)
Weight loss	36/52 (69%)	18/27 (67%)	18/25 (72%)
Lymphadenopathy	3/53 (5.7%)	0/27 (0%)	3/26 (12%)
Skin lesion	9/52 (17%)	4/26 (15%)	5/26 (19%)
Oral mucosa lesion	8/53 (15%)	3/27 (11%)	5/26 (19%)
Genital mucosa lesion	3/53 (5.7%)	1/27 (3.7%)	2/26 (7.7%)
Edema	9/53 (17%)	6/27 (22%)	3/26 (12%)
Oligoanuria	7/52 (13%)	2/27 (7.4%)	5/25 (20%)
Respiratory symptoms	42/53 (79%)	21/27 (78%)	21/26 (81%)
Respiratory failure	18/52 (35%)	7/27 (26%)	11/25 (44%)
Diarrhea	21/53 (40%)	10/27 (37%)	11/26 (42%)
Hepatomegaly	3/52 (5.8%)	1/27 (3.7%)	2/25 (8.0%)
Splenomegaly	1/52 (1.9%)	0/27 (0%)	1/25 (4.0%)
Gastrointestinal bleeding	3/52 (5.8%)	2/27 (7.4%)	1/25 (4.0%)
Focal neurological deficit	3/53 (5.7%)	2/27 (7.4%)	1/26 (3.8%)
Nausea	19/47 (40%)	10/23 (43%)	9/24 (38%)
Vomiting	19/48 (40%)	10/23 (43%)	9/25 (36%)
**Laboratory findings**			
Albumin (g/dL)	2.45 (2.00, 3.30), [54]	2.55 (2.25, 3.25), [28]	2.25 (1.50, 3.30), [26]
Calcium	7.70 (6.40, 9.30), [42]	8.05 (6.30, 9.80), [22]	7.50 (6.55, 8.90), [20]
Creatinine (mg/dL)	1.40 (0.80, 4.95), [56]	2.55 (1.00, 5.60), [30]	1.25 (0.60, 3.60), [26]
Hematocrit (%)	24 (20, 29), [58]	26 (20, 31), [31]	23 (20, 27), [27]
Hemoglobin (g/dL)	7.74 (6.46, 9.44), [58]	8.11 (6.77, 9.72), [31]	7.39 (6.42, 9.44), [27]
MCV (fL)	85 (80, 92), [58]	84 (82, 98), [31]	86 (80, 91), [27]
Ferritin			
<=3000	2/15 (13%)	2/6 (33%)	0/9 (0%)
>3000	13/15 (87%)	4/6 (67%)	9/9 (100%)
High C-reactive protein category			
<6.5	5/40 (13%)	4/22 (18%)	1/18 (5.6%)
>=6.5	35/40 (88%)	18/22 (82%)	17/18 (94%)
White blood cells (/mm³)	8,020 (3,081, 17,640), [58]	7,776 (3,604, 19,470), [31]	8,264 (2,244, 17,600), [27]
Neutrophils (%)	89 (82, 94), [56]	90 (81, 95), [30]	89 (84, 93), [26]
Lymphocytes (%)	5 (3, 8), [57]	4 (2, 8), [30]	5 (3, 10), [27]
Platelets (/mm³)	75,035 (36,690, 161,400), [58]	62,860 (33,160, 161,200), [31]	101,300 (36,690, 204,500), [27]
Alkaline phosphatase (IU/L)	677 (370, 1,206), [53]	562 (363, 963), [28]	794 (370, 1,968), [25]
LDH (U/L)	813 (572, 1,670), [56]	760 (498, 1,661), [30]	844 (600, 1,708), [26]
AST (IU/L)	71 (41, 130), [56]	85 (39, 111), [30]	60 (42, 130), [26]
ALT (IU/L)	65 (23, 103), [56]	67 (32, 99), [30]	48 (22, 106), [26]
Total bilirubin (mg/dL)	0.81 (0.51, 1.87), [51]	0.66 (0.51, 1.86), [26]	0.83 (0.58, 1.98), [25]
Direct bilirubin (mg/dL)	0.39 (0.21, 1.26), [50]	0.42 (0.21, 1.05), [25]	0.36 (0.21, 1.55), [25]
Indirect bilirubin (mg/dL)	0.47 (0.27, 0.82), [50]	0.48 (0.25, 0.82), [25]	0.46 (0.28, 0.64), [25]

**Abbreviations:** ART, antiretroviral therapy; ICU, intensive care unit; LDH, lactate dehydrogenase; AST, aspartate aminotransferase; ALT, alanine aminotransferase; MCV, mean corpuscular volume; PML, progressive multifocal leukoencephalopathy; IQR, interquartile range.

Histoplasmosis was identified in 27/59 (45.8%) patients. Compared with those without histoplasmosis, affected individuals more frequently reported alcohol use (72% vs 22%) and recreational drug use (39% vs 12%), had a shorter median time since HIV diagnosis (3 vs 57 months), and more often presented with lower hemoglobin concentrations and ferritin levels >3000 ng/mL. Additional demographic, clinical, and laboratory characteristics according to histoplasmosis status are presented in Tables 1 and 2.

**Table 2:** Detection of *Histoplasma* and classification of infection.

	Liver Culture/PCR/ Real-time PCR / Ct qPCR/ Histopathology	Brain Culture/PCR/ Real-time PCR/ Ct qPCR/ Histopathology	Lung Culture/PCR/ Real-time PCR/ Ct qPCR/ Histopathology	Blood Culture/PCR/ Real-time PCR/ Ct qPCR/ Histopathology	CSF Culture/PCR/ Real-time PCR/ Ct qPCR/ Histopathology	Culture (overall)	PCR (overall)	qPCR (overall)	Histopathology (overall)	Classifications	Cause of death	Clinical interpretation of the cause of death
1	-/ + /-/-/-	-/ + /-/-/-	-/-/-/-/-	-/-/-/-	-/-/-/-	Negative	Positive	Negative	Negative	Multi-organ detection	Kaposi’s Sarcoma	Not related
2	-/-/-/-/-	-/-/-/-/-	-/-/-/-/-	-/-/-/-	-/ + /-/-	Negative	Positive	Negative	Negative	Single-organ/body fluid detection	Undetermined	Uncertain
3	-/ + / + /38/-	-/ + /-/-/-	+/ + / + /39/-	-/-/-/-	-/-/-/-	Positive	Positive	Positive	Negative	Multi-organ detection	Bacterial pneumonia	Uncertain
4	+/ + / + /26/+	-/-/ + /41/-	+/ + / + /22/+	+/-/ + /35	+/-/-/-	Positive	Positive	Positive	Positive	Multi-organ detection	Disseminated histoplasmosis	Primary
5	-/-/-/-/-	-/-/-/-/-	-/-/-/-/-	-/-/-/-	-/-/-/-	Negative	Negative	Negative	Negative	Single-organ/body fluid detection	Undetermined	Not related
6	-/-/-/-/-	-/-/-/-/-	-/-/-/-/-	+/-/-/-	-/-/-/-	Positive	Negative	Negative	Negative	Single-organ/body fluid detection	Undetermined	Uncertain
7	-/-/-/-/+	-/-/-/-/-	-/-/-/-/-	-/-/-/-	-/-/-/-	Negative	Negative	Negative	Positive	Single-organ/body fluid detection	Tuberculosis and Pneumonia	Contributory
8	-/-/-/-/-	-/-/-/-/-	-/-/-/-/+	-/-/-/-	-/-/-/-	Negative	Negative	Negative	Positive	Lung-restricted detection	Diffuse alveolar damage	Contributory
9	+/ + / + /41/-	+/-/ + /47/-	-/ + / + /31/-	-/-/ + /41	+/ + / + /43	Positive	Positive	Positive	Negative	Multi-organ detection	Diffuse alveolar damage	Contributory
10	-/-/-/-/-	-/-/-/-/-	-/-/-/-/-	-/-/-/-	-/-/ + /43	Negative	Negative	Positive	Negative	Single-organ/body fluid detection	Intra-alveolar hemorrhage	Not related
11	+/ + / + /35/+	-/-/-/-/-	-/ + / + /31/+	-/-/-/-	-/-/-/-	Positive	Positive	Positive	Positive	Multi-organ detection	Pulmonary histoplasmosis, pulmonary cytomegalovirus and hepatic histoplasmosis	Primary
12	-/ + / + /33/-	-/-/-/-/-	-/-/-/-/-	-/-/-/-	-/-/-/-	Negative	Positive	Positive	Negative	Single-organ/body fluid detection	Fungal lung disease and aspiration pneumopathy	Uncertain
13	-/-/-/-/-	-/-/-/-/-	-/-/-/-/-	-/ + /-/-	-/-/-/-	Negative	Positive	Negative	Negative	Single-organ/body fluid detection	Pneumopathy Undetermined	Not related
14	-/-/-/-/-	-/-/-/-/-	-/-/-/-/-	-/ + /-/-	-/-/-/-	Negative	Positive	Negative	Negative	Single-organ/body fluid detection	Undetermined	Not related
15	-/-/-/-/-	-/-/-/-/-	-/-/-/-/-	-/ + /-/-	-/-/-/-	Negative	Positive	Negative	Negative	Single-organ/body fluid detection	Acute respiratory failure	Not related
16	+/ + / + /33/-	+/-/-/-/-	+/ + / + /28/-	+/ + / + /-	-/-/-/-	Positive	Positive	Positive	Negative	Multi-organ detection	Pulmonary histoplasmosis, hepatic histoplasmosis	Primary
17	-/-/-/-/-	-/-/-/-/-	-/-/-/-/-	-/ + /-/-	-/-/-/-	Negative	Positive	Negative	Negative	Single-organ/body fluid detection	Pulmonary cryptococcosis, pulmonary Kaposi’s sarcoma	Not related
18	-/-/-/-/-	-/-/-/-/-	-/-/-/-/-	+/ + /-/-	-/-/-/-	Positive	Positive	Negative	Negative	Single-organ/body fluid detection	Respiratory failure, bacterial pneumonia, pneumocystosis	Contributory
19	-/-/-/-/-	-/-/-/-/-	-/-/-/-/-	-/ + /-/-	-/-/-/-	Negative	Positive	Negative	Negative	Single-organ/body fluid detection	Undetermined	Not related
20	-/-/-/-/-	-/-/-/-/-	-/-/-/-/-	-/ + /-/-	-/-/-/-	Negative	Positive	Negative	Negative	Single-organ/body fluid detection	Undetermined, pneumopathy	Not related
21	-/-/-/-/-	-/-/-/-/-	-/-/-/-/-	-/ + /-/-	-/-/-/-	Negative	Positive	Negative	Negative	Single-organ/body fluid detection	Cryptococcal encephalitis and bacterial pneumonia	Not related
22	-/-/-/-/-	-/-/-/-/-	-/-/-/-/-	-/ + /-/-	-/-/-/-	Negative	Positive	Negative	Negative	Single-organ/body fluid detection	Pneumocystis	Not related
23	-/-/-/-/-	-/-/-/-/-	-/-/-/-/-	-/ + /-/-	-/-/-/-	Negative	Positive	Negative	Negative	Single-organ/body fluid detection	Undetermined	Not related
24	+/ + / + /44/-	+/-/-/-/-	+/ + / + /45/-	+/-/-/-	+/-/ + /48	Positive	Positive	Positive	Negative	Multi-organ detection	Pneumonia	Contributory
25	-/-/-/-/-	-/-/-/-/-	-/-/-/-/-	-/ + /-/-	-/-/-/-	Negative	Positive	Negative	Negative	Single-organ/body fluid detection	Disseminated cryptococcosis,	Not related
26	-/-/-/-/+	-/-/-/-/-	-/ + /-/-/+	-/ + /-/-	-/-/-/-	Negative	Positive	Negative	Positive	Multi-organ detection	TB pulmonary and hepatic tuberculosis	Contributory
27	-/ + / + /31/-	-/-/ + /26/-	-/ + / + /37/-	+/NA/NA/0	-/-/ + /40	Negative	Positive	Positive	Negative	Multi-organ detection	Histoplasmosis, Pulmonary cryptococcosis	Primary

**Abbreviations:** PCR, polymerase chain reaction; qPCR, quantitative polymerase chain reaction; CSF, cerebrospinal fluid.

**Symbols:** + positive; − negative; NA not available.

**Other Samples evaluated**: 4** Ileum +/+/+ (49); Ascitic fluid -/-/+ (33) and 5*Pleural fluid -/-/+ (40).

### Tissue distribution of *Histoplasma capsulatum*

*H. capsulatum* was detected in 27 of 59 patients (45.8%). According to the predefined classification, 9 patients (33.3%) had multi-organ detection of *H. capsulatum*, 1 had lung-restricted detection, and 17 had single-organ/body-fluid detection. Liver and lungs were the most frequently involved organs, with fungal detection in 9/27 (33.3%) and 8/27 (29.6%) patients, respectively. Brain tissue, blood, and cerebrospinal fluid were less frequently involved.

Distinct patterns of tissue involvement were observed according to the predefined tissue-distribution categories. Multi-organ with frequent involvement of the liver (9/9, 100%), lungs (8/9, 88.9%), blood (6/9, 66.7%), and cerebrospinal fluid (4/9, 44.4%). In contrast, single organ/body fluid detection was predominantly detected locally, most commonly in blood (12/17, 70.6%), whereas liver and cerebrospinal fluid involvement were less frequent (2/17, 11.8% each). The single patient with Lung-restricted detection had fungal detection restricted to the lungs (Table 3).

**Table 3:** Distribution of *H. capsulatum* detection by tissue according to the predefined tissue-distribution categories.

Organ	Multi-organ detection (n = 9)	Lung-restricted detection (n = 1)	Single-organ/body fluid detection (n = 17)
Lung (any method*)	8/9 (88.9%)	1/1 (100%)	0/17 (0%)
Liver (any method*)	9/9 (100%)	0/1 (0%)	2/17 (11.7%)
Brain (any method*)	3/9 (33.3%)	0/1 (0%)	0/17 (0%)
Blood (any method*)	6/9 (66.7%)	0/1 (0%)	12/17 (70.5%)
CSF (any method*)	4/9 (44.4%)	0/1 (0%)	2/17 (11.7%)

*Any positive method includes culture, qPCR, histopathology, or a combination thereof. CSF: cerebrospinal fluid. Missing data were recorded as not available.

### Contribution of laboratory methods to tissue detection of *H. capsulatum*

Molecular assays detected fungal DNA in 23 of 27 patients (85.2%), whereas fungal culture and histopathology identified fungal infection in 8 (29.6%) and 5 (18.5%) patients, respectively. Culture most frequently yielded positive results from liver (5/27) and blood (6/27) samples, whereas PCR-based methods detected fungal DNA across multiple tissues. Molecular methods detected fungal DNA in tissues from 16 patients, while their corresponding culture samples were negative.

In several instances, molecular detection occurred in the absence of fungal growth in culture, particularly in samples with low fungal DNA burden. qPCR amplification was observed in multiple organs. Median Ct values were lower in lung tissue (40.3, range 37.0–45.0) and liver (41.0, range 38.0–44.0) than in cerebrospinal fluid (43.5, range 40.0–48.0) and brain tissue (44.0, range 41.0–47.0), indicating stronger molecular amplification signals in those organs (Table 4).

**Table 4:** Detection of *Histoplasma capsulatum* across different organs and sample types using culture, PCR, qPCR, and histopathology.

Organ	Culture	PCR	qPCR	Histopathology	qPCR Ct values
	[n/N (%)]	[n/N (%)]	[n/N (%)]	[n/N (%)]	(median, range)
**Main Organs (N = 27)**					
**Lung**	4/27 (14.8%)	8/27 (29.6%)	7/27 (25.9%)	4/27 (14.8%)	40.3 (37.0–45.0)
**Liver**	5/27 (18.5%)	9/27 (33.3%)	8/27 (29.6%)	4/27 (14.8%)	41.0 (38.0–44.0)
**Brain**	3/27 (11.1%)	2/27 (7.4%)	3/27 (11.1%)	0/27 (0.0%)	44.0 (41.0–47.0)
**Blood**	6/27 (22.2%)	13/27 (48.1%)	3/27 (11.1%)	—	41
**CSF**	3/27 (11.1%)	2/27 (7.4%)	4/27 (14.8%)	—	43.5 (40.0–48.0)
**Other samples evaluated**					
**Ileum**	1/1 (100.0%)	1/1 (100.0%)	1/1 (100.0%)	—	49
**Ascitic Fluid**	0/1 (0.0%)	0/1 (0.0%)	1/1 (100.0%)	—	33
**Pleural Fluid**	0/1 (0.0%)	0/1 (0.0%)	1/1 (100.0%)	—	40

**Abbreviations:** PCR, polymerase chain reaction; qPCR, quantitative polymerase chain reaction; CSF, cerebrospinal fluid; Ct, cycle threshold.

**Footnote:** Molecular detection was defined as positivity by conventional PCR and/or qPCR. Ct values were generated exclusively from qPCR assays and were interpreted descriptively as indicators of detectable fungal DNA signal intensity rather than direct measures of viable fungal burden or disease severity. Amplification signals near the assay’s technical detection limit should be interpreted cautiously.

### Histopathological findings

Histopathological examination demonstrated small intracellular and extracellular yeast forms morphologically compatible with *H. capsulatum*, measuring approximately 2–4 μm. GMS and PAS stains enhanced the visualization of fungi.

**Fig 1:**
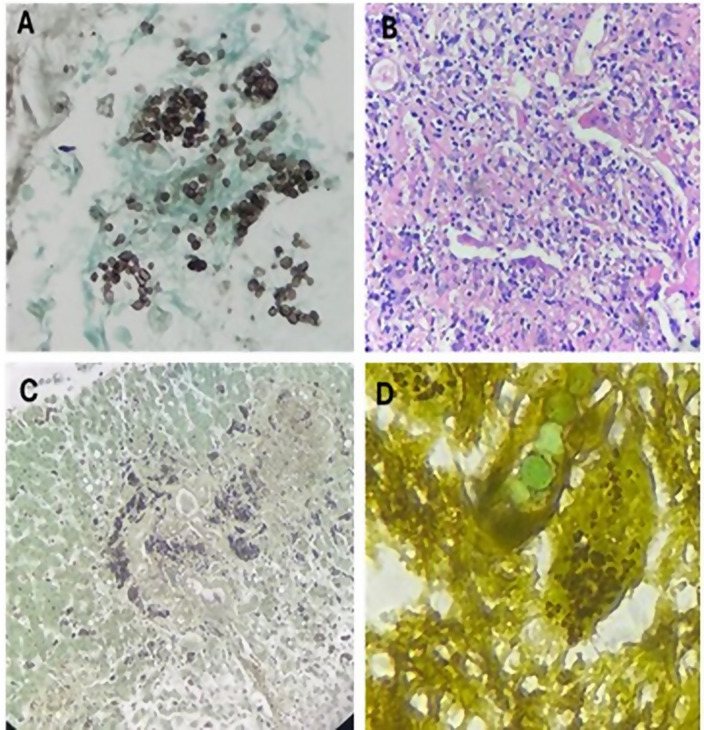
Autopsy findings in the lungs (A and B), liver (C) and brain (D) of patients with histoplasmosis. **A)** Histoplasma inside the alveolar spaces. The yeasts are small, oval, with a clear halo surrounding them, which is compatible with *Histoplasma capsulatum*. They are isolated and not associated with inflammatory reactions (Grocott silver methenamine stain). Magnification = 400X. **B)** Lung tissue shows areas of necrosis and granulomatous reaction composed of epithelioid cells (Periodic acid-Schiff stain). Magnification = 200X **C)** Granulomas and histoplasmosis in the portal space in liver tissue (Grocott silver methenamine stain). Magnification = 100X. **D)** Brain with histoplasma without granulomatous reaction (Grocott silver methenamine stain). Magnification = 400X.

In the lungs, organisms were observed within alveolar spaces and macrophages, with variable inflammatory responses ranging from minimal inflammation to necrotizing granulomatous lesions. Liver specimens showed granulomatous inflammation centered on portal tracts, with fungal organisms located within macrophages and portal granulomas. Brain tissue demonstrated scattered yeast forms without a significant inflammatory or granulomatous response (Fig 1).

## Discussion

Histoplasmosis remains one of the leading neglected infectious diseases and an important cause of mortality among PLHIV in Latin America [35]. In this study, we characterized the postmortem tissue distribution of *H. capsulatum* in deceased PLHIV using MITS integrated with conventional microbiological, histopathological, and molecular methods. Histoplasmosis was identified in 27 of 59 patients (45.8%), and one-third of these cases (33.3%) showed multi-organ fungal detection, underscoring the substantial burden of fatal histoplasmosis among individuals with advanced HIV disease in the Brazilian Amazon. By integrating complementary diagnostic approaches, our study provided a more comprehensive assessment of tissue involvement than fungal culture alone, particularly in patients with disseminated disease affecting multiple organs.

The observed frequency of histoplasmosis confirms previous reports highlighting the substantial contribution of this infection to AIDS-related mortality in Latin America. Among the 59 evaluated individuals, 45.8% showed evidence of the disease, and in 16.9% of the cases, histoplasmosis was considered the primary cause of death. These results are consistent with data from Guatemala [36], Colombia [37], French Guiana [38], and the Brazilian Amazon [14,39]. Although the prevalence observed in our study was slightly higher than that reported by Rakislova et al. [14], who identified histoplasmosis in 38% of HIV-associated deaths in the Brazilian Amazon, the proportion of deaths directly attributed to histoplasmosis was lower in that series. On the other hand, mortality in this study was higher than that reported by Souza et al. [40], possibly reflecting the low frequency of antiretroviral therapy at hospital admission and the profound immunosuppression observed in our patients.

Unlike previous studies that focused primarily on the clinical characteristics, therapeutic outcomes, mortality, coinfections, or diagnostic performance of disseminated histoplasmosis, our research systematically characterizes the anatomical distribution of *H. capsulatum* across multiple tissues through MITS integrated with fungal culture, histopathology, conventional PCR, and quantitative PCR. This integrated approach provides novel pathological evidence regarding tissue involvement in fatal HIV-associated histoplasmosis and highlights the importance of combining complementary laboratory methods to enhance the characterization of disseminated fungal infections.

The strength of the study is the use of MITS, which enabled a systematic postmortem evaluation of tissue involvement. Although MITS does not provide the comprehensive anatomical view of a complete autopsy, previous validation studies have demonstrated good agreement between the two approaches in determining infectious causes of death, especially in low- and middle-income settings [19,41,42]. Beyond its value as a pathological research tool, MITS has important implications for public health surveillance [41,43]. Facilitating standardized postmortem tissue collection with relatively simple infrastructure can promote recognition of fatal histoplasmosis and other opportunistic infections, strengthen mortality surveillance, and generate more accurate estimates of disease burden in regions where conventional autopsies and advanced diagnostic methods remain limited [18,43–46].

From a clinical perspective, histoplasmosis manifests with non-specific symptoms, including fever, weight loss, respiratory symptoms, and diarrhea, which resemble those of tuberculosis and other opportunistic infections [7,47–49]. Consequently, only a minority of patients received a diagnosis and antifungal therapy prior to death. Similar results have been observed in other endemic regions [35,49], highlighting the persistent underdiagnosis of histoplasmosis among PLHIV. Although the disease has historically been considered predominantly a Latin American problem, growing evidence indicates that it is also a relevant, yet underrecognized, cause of HIV-associated morbidity and mortality in Sub-Saharan Africa and Southeast Asia [50–54]. Limited access to *Histoplasma* antigen detection, molecular diagnostics, and pathological investigations contributes to delayed diagnosis, frequent confusion with tuberculosis, and the underestimation of the disease burden [10,35,55,56]. Thus, the challenges identified in the Brazilian Amazon reflect a broader global reality, in which disseminated histoplasmosis remains a neglected opportunistic infection, despite high mortality among immunocompromised individuals [57].

The liver and lungs had the highest positivity rates for *H. capsulatum*, followed by blood and cerebrospinal fluid. Multiorgan detection was common, whereas detection restricted to the lungs was relatively rare. These findings expand current knowledge on tissue involvement in fatal HIV-associated histoplasmosis and corroborate previous postmortem observations made in the Brazilian Amazon [14]. Although the relatively small number of histoplasmosis cases precludes definitive conclusions regarding preference for specific organs, the observed distribution provides important pathological evidence concerning the anatomical dissemination of *H. capsulatum* in advanced stages of HIV infection.

The predominance of fungal detection in the lungs and liver is consistent with the established pathogenesis of DH. Following inhalation, the lungs constitute the primary portal of infection, where microconidia transform into yeast forms and are rapidly phagocytosed by alveolar macrophages [58,59]. Instead of being eliminated, *H. capsulatum* survives and replicates intracellularly, thereby interfering with phagolysosomal maturation and evading the host’s antimicrobial mechanisms [60,61]. Infected macrophages then disseminate through the lymphatic system and bloodstream to organs rich in mononuclear phagocyte system cells, such as the liver, spleen, bone marrow, and lymph nodes [62–65]. The high frequency of hepatic involvement observed in our study likely reflects the abundance of resident macrophages (Kupffer cells), which provide a favorable niche for fungal intracellular persistence, especially in advanced HIV-associated immunosuppression.

The heterogeneous patterns of tissue involvement indicate that DH does not constitute a uniform pathological process. Although the cross-sectional postmortem design precludes conclusions regarding disease progression, the three predefined patterns of tissue distribution, multiorgan detection, detection restricted to the lungs, or single organ/body fluid detection, may reflect different stages or trajectories of fungal dissemination [1,66]. Detection restricted to the lungs may indicate infection confined to the primary site of inoculation or incomplete dissemination, whereas multiorgan detection is compatible with widespread hematogenous dissemination mediated by infected macrophages [1,2,66,67]. In turn, detection limited to a single extrapulmonary organ or body fluid may indicate initial dissemination, localized persistence, or heterogeneous fungal distribution that is only partially captured by MITS [68,69]. These hypotheses require validation in prospective studies with antemortem sampling and longitudinal clinical follow-up.

Although our observations are based exclusively on postmortem samples, they provide valuable insights into future research and diagnostic strategies. The predominance of fungal detection in the liver and lungs suggests that these organs may be higher-yield targets when tissue collection is clinically indicated, especially in settings lacking access to *Histoplasma* antigen detection or advanced molecular diagnostics. However, such findings should not be interpreted as direct clinical recommendations but rather as a pathological framework to guide prospective studies evaluating the diagnostic yield of different biopsy sites and to refine sampling protocols for antemortem investigations and standardized MITS procedures. Integrating anatomical distribution with complementary laboratory methods may ultimately contribute to the development of evidence-based diagnostic strategies for DH in endemic and resource-limited environments.

The conventional diagnosis of histoplasmosis relies on fungal culture and the histopathological demonstration of yeast forms using PAS or GMS stains [70]. However, both methods present significant limitations. Culture is slow, often requiring weeks for fungal growth, has reduced sensitivity in disseminated disease [16,70], and demands Biosafety Level 3 laboratory infrastructure due to the infectious nature of H. capsulatum [71]. Histopathological confirmation can also be limited by a low fungal burden or the small tissue fragments obtained through MITS. In our series, histopathology confirmed only a minority of cases, despite molecular detection across multiple tissues, highlighting the complementary rather than competing role of these diagnostic approaches. Together, the focal distribution of the fungus, prior antifungal therapy, and advanced immunosuppression can reduce microscopic visualization, while fungal DNA remains detectable by molecular methods.

Molecular methods should be considered complementary diagnostic tools, rather than substitutes. In this postmortem series, PCR-based assays detected fungal DNA in culture-negative tissues, expanding the characterization of tissue involvement without implying diagnostic superiority. While fungal culture confirms the presence of viable organisms and histopathology provides direct evidence of tissue invasion and host response, molecular methods increase the likelihood of detecting fungal DNA in samples with low burden or after prior exposure to antifungals [72–74]. The integration of these approaches allowed for a more complete assessment of the anatomical distribution of H. capsulatum than would be possible with any single method.

Although some qPCR-positive samples had Ct values close to the analytical limit of detection, these results should be interpreted with caution as indicative of fungal DNA presence rather than definitive confirmation of viable organisms or active infection. However, when analyzed alongside culture, histopathology, tissue distribution, and the overall pathological context, molecular methods strengthened the characterization of disseminated histoplasmosis, identifying DNA in tissues that would otherwise have remained undiagnosed. Since the evaluation of diagnostic accuracy was not the focus of this descriptive postmortem study, these findings should be viewed primarily as complementary evidence of tissue involvement.

The contribution of *H. capsulatum* to death was evaluated based on pathological findings obtained through MITS. Although this technique provides a standardized assessment of tissue involvement, it does not fully capture the clinical course prior to death and therefore should be interpreted as a pathological analysis of histoplasmosis’s role rather than a definitive determination of the clinical cause of death. Even so, the high proportion of patients for whom histoplasmosis was considered a primary or contributing cause highlights the significant impact of this neglected mycosis among PLHIV in endemic areas.

Beyond the pathological implications, this study is relevant to public health. The high frequency of histoplasmosis identified among deceased PLHIV reinforces the persistent underdiagnosis of the disease and indicates the need to strengthen diagnostic capacity in HIV care programs. Expanding access to *Histoplasma* antigen detection, molecular diagnostics, and standardized postmortem investigations can facilitate early diagnosis, improve mortality surveillance, and provide more accurate estimates of the burden of HIV-associated histoplasmosis. These results support efforts to integrate neglected fungal diseases into HIV control programs and public health surveillance systems, especially in low- and middle-income countries, where late diagnosis remains a relevant factor in preventable mortality.

This study has significant limitations. The relatively small sample size, the single-center design, and the exclusive inclusion of deceased PLHIV introduce a selection bias towards cases of advanced disease and fatal clinical manifestations, which restrict the generalizability of the results to other endemic settings. Therefore, the patterns observed in tissue distribution should not be extrapolated to patients diagnosed in early stages of infection, those with less severe immunosuppression, or individuals who respond adequately to antifungal treatment. Furthermore, as this is a post-mortem investigation, it was not possible to establish longitudinal clinical correlations between fungal tissue distribution, disease progression, and therapeutic response. Detection of *Histoplasma* antigen, an important diagnostic tool for disseminated histoplasmosis, was also unavailable for comparison with microbiological, histopathological, and molecular findings.

Other methodological limitations also deserve consideration. Several positive samples showed Ct values close to the analytical detection limit, requiring careful interpretation, as this may indicate a low fungal DNA load or post-mortem DNA degradation rather than viable organisms or active infection in the tissues. Post-mortem autolysis, prior antifungal use, and the limited amount of tissue obtained by MITS may also have interfered with fungal detection. Variability in access to different organs may have impacted sample representativeness. The absence of an independent gold standard prevented a formal evaluation of diagnostic performance. Therefore, the proposed categories for tissue distribution should be understood as a pathological reference for HIV-associated histoplasmosis in fatal cases, and not as clinical classifications or guidelines for sample collection. Prospective, multicenter studies integrating *Histoplasma* antigen detection, longitudinal clinical data, and standardized tissue sampling are needed to validate and improve these results.

## Conclusion

The combination of MITS with conventional microbiological, histopathological, and molecular methods enabled comprehensive characterization of the postmortem distribution of *H. capsulatum* in PLHIV with advanced disease. By integrating complementary diagnostic approaches, this study expands current knowledge on fungal dissemination, provides novel pathological evidence regarding tissue involvement in fatal HIV-associated histoplasmosis, and supports the use of MITS as a viable platform for pathological investigations and mortality surveillance in endemic areas. Although these findings should not be directly extrapolated to clinical practice, they establish a solid foundation for future prospective studies evaluating tissue-based diagnostic strategies and reinforce the potential of MITS to strengthen surveillance of neglected fungal diseases, support evidence-based public policies, and reduce HIV-associated mortality.

## Supporting information

S1 Table Clinical and demographic characteristics at hospital admission of people living with HIV (PLHIV) included in the MITS study. (DOCX)

S2 Table Characteristics at moment of HIV diagnosis. (DOCX)
