# Supplementary material for: Tissue distribution of Histoplasma capsulatum in deceased people living with HIV assessed by minimally invasive tissue sampling: An autopsy-based case series in the Brazilian Amazon
Source: PLoS Negl Trop Dis. 2026 Aug 6;20(8):e0014545. doi: 10.1371/journal.pntd.0014545 (PMC13446691; doi:10.1371/journal.pntd.0014545)
Supplement: Supplementary file 1 [file pntd.0014545.s003.pdf]

The manuscript **PNTD-D-25-01973**, entitled *Parasitic Burden of Histoplasma capsulatum in Different Tissues Collected through Minimally Invasive Tissue Sampling in Deceased Patients with HIV/AIDS from the Brazilian Amazon*, is scientifically sound. The study design and setting are informative and present several strengths.

The Introduction is scientifically solid and well structured, with strong regional relevance and a clear public health message. With moderate tightening and sharper articulation of the knowledge gap, it would be well positioned for submission to an international infectious diseases journal.

The institutional identification is appropriate, and the setting is clearly described, including the hospital's tertiary profile and its regional relevance. The inclusion of prior epidemiological data (34% histoplasmosis among autopsied patients) strengthens the study rationale. The section describing the patients and ethical aspects presents clear inclusion criteria. Ethics approval is properly mentioned with the CAAE number, and the postmortem interval (<48 h) is defined, which is methodologically relevant.

There is a clear description of MITS use, a detailed list of tissues and fluids collected, a well-documented sample prioritization strategy, and adequate chain-of-custody control. The data source is clearly identified (electronic medical records), which enhances transparency. The temporal framework is defined, indicating that information was collected at HIV diagnosis, prior to hospitalization, and during hospitalization. This longitudinal approach suggests an attempt to capture the full clinical trajectory of the patients.

The mycological diagnosis describes appropriate and well-specified culture media and suitable incubation temperatures, with a prolonged incubation period (30–45 days) consistent with *Histoplasma*. Classic morphological identification methods are also described. The methodology is solid and based on previously validated protocols, with appropriate use of molecular and histopathological techniques.

The data presented in the Results section are clinically important and potentially impactful, particularly given the high prevalence of histoplasmosis in advanced HIV. The Discussion is clinically relevant and regionally important. It presents strong epidemiological framing (Amazon region, PLHIV, mortality), good integration of diagnostic methods (culture, histopathology, qPCR, MITS), clear public health implications, appropriate comparison with Latin American literature, and a practical diagnostic algorithm.

However, several points require improvement:

1) Introduction section

There is some conceptual redundancy, minor inaccuracies or overgeneralizations, and overstatement in certain claims. The study rationale also requires sharper refinement. For instance, in the study aim paragraph (Page 10, Line 126), the authors state: "identifying which tissues have the most significant potential for clinical sampling among alive patients…"

This should be revised because the authors are using postmortem MITS samples, and translating these findings directly to living patients requires caution. A more appropriate formulation would be: "to inform potential prioritization of tissues for clinical sampling…"

Additionally, the objective includes correlating fungal load, identifying optimal tissues, guiding clinical sampling, improving timeliness, and focusing on immunosuppressed individuals. This is overly broad. Please consider simplifying to:

- **Primary objective:** Correlate fungal load with tissue distribution.
- **Secondary implication:** Inform diagnostic strategies.

High-impact journals generally prefer focused objectives.

The flow between paragraphs could also be smoother, and language tightening is required in several places, such as:

- Page 9, Line 101: *Histoplasma* should be italicized.
- Page 9, Line 102: "through public health systems" → change to "within public health systems."
- Page 10, Line 125: "to correlate histoplasma load" → capitalize and italicize *Histoplasma*.
- Page 10, Line 127: "among alive patients" → change to "among living patients."
- Some sentences exceed 35–40 words; consider splitting them for clarity.

2) Study design lacks specificity

Page 10, Line 133: "This study is observational in a series of cases" is grammatically imprecise.

The appropriate terminology would be:

- "observational case series study"
  or
- "observational autopsy-based case series."

3) Epidemiological justification could be better connected

Page 11, Lines 138–140: The 34% finding is relevant, but a sentence explicitly linking this result to the need for the present study is missing.

4) Incorrect geographic location

Page 11, Line 136: Manaus is located in the Northern Region of Brazil, not in the "Northeast of the Amazonian State." This should be corrected to avoid a geographic error.

5) Exclusion criteria not described

Please clarify whether cases with advanced decomposition, known prior coinfections, or marked autolysis were excluded.

6) HIV diagnosis not defined

It would be important to specify whether HIV infection was confirmed by laboratory criteria or based solely on clinical records.

7) "Randomly punctured the tissues"

This wording may raise methodological concerns. Random sampling could reduce diagnostic representativeness. Ideally, the manuscript should indicate:

- Standardized number of punctures per organ
- Approximate depth of sampling
- Whether systematic anatomical orientation was followed

8) Reproducibility

To increase reproducibility, please include information about:

- Number of fragments collected per organ
- Minimum volume obtained
- Standardization across cases

9) Cross-contamination control

Given that molecular and mycological analyses were performed, it is important to mention:

- Needle replacement between organs
- Anti-contamination measures
- Use of negative controls

10) Molecular transport conditions

Page 12, Line 160: "Kept on cold chain" is vague. Please specify the exact temperature (later mentioned as 2–8 °C) and the maximum time until processing.

11) Clinical Information section

The section does not specify which demographic, epidemiological, or clinical variables were extracted. For methodological transparency and reproducibility, please clarify whether the following were collected:

- Age and sex
- HIV-related parameters (CD4 count, viral load, ART use)

- Opportunistic infections
- Prior antifungal therapy
- Comorbidities
- Laboratory parameters (e.g., liver enzymes, hematologic profile)

The section also does not specify how HIV diagnosis was confirmed (laboratory confirmation vs. clinical record), which is particularly relevant in autopsy-based studies.

The term "epidemiological information" is broad and undefined. It would be helpful to specify whether place of residence (urban vs. rural), occupational exposure, travel history, or environmental exposure risks were collected.

Additionally, there is no mention of standardized data collection or double review procedures. Please clarify:

- Whether data extraction followed a standardized case report form
- Whether more than one reviewer performed data abstraction
- How discrepancies were resolved
- Whether missing data were handled systematically

Given that retrospective record review is subject to information bias, this should be addressed.

Overall, the section is clear but overly concise and methodologically underdeveloped. Expanding it would significantly improve scientific rigor and reproducibility.

12) Mycological analysis

It is unclear whether fungal identification was based solely on morphology and thermal dimorphism testing or whether molecular confirmation was performed. Additionally, to strengthen methodological robustness, please describe:

- Time to growth considered positive
- Whether repeat cultures were performed
- Biosafety level for *Histoplasma* handling (BSL-2 or BSL-3)

13) Molecular methods – analytical performance

The manuscript does not report:

- Limit of detection (LOD)
- Analytical sensitivity and specificity
- Amplification efficiency
- Validation in autopsy specimens (if different from the original validation study)

Including these parameters would strengthen confidence in diagnostic performance.

Please also mention potential cross-reactivity with phylogenetically related fungi and clarify how Ct values were interpreted.

14) Histopathological criteria

The morphological criteria used to define *Histoplasma* spp. in tissue are not described (e.g., yeast size, intracellular location, narrow-based budding). Explicit criteria would enhance reproducibility.

15) Results section

The Results section requires statistical clarification, table restructuring, and removal of overinterpretation. Essential corrections include:

- Replace all "median ± SD" with appropriate statistical format.
- Clarify denominators for all percentages.
- Remove unsupported sensitivity/specificity claims.
- Define qPCR positivity threshold.
- Reformat Table 1 with a clear legend and standardized notation.
- Clearly explain classification criteria.

16) Discussion section

Lines 333–356 are conceptually strong but repetitive. The authors repeat:

- Nonspecific symptoms
- Overlap with tuberculosis
- Limited diagnostics in Latin America
- Regional lack of access

Please condense this into one cohesive paragraph emphasizing clinical overlap, structural diagnostic limitations, and the consequences of delayed or missed treatment.

The interpretation of qPCR performance is the most critical methodological section. The statement "qPCR performed remarkably well… demonstrating superiority…" requires moderation because:

- The study uses postmortem tissue (MITS).
- There is no gold-standard comparator for all cases.
- Molecular detection does not necessarily indicate viable infection.
- Ct >38 ambiguity is acknowledged but not fully explored.

To strengthen scientific rigor, please explicitly address:

- Risk of DNA persistence postmortem
- Risk of environmental contamination
- Lack of standardized Ct cut-offs
- Absence of antigen testing in most cases

Instead of "superiority," consider "demonstrated higher analytical sensitivity compared with culture…"

Regarding Ct interpretation, please clarify:

- Was Ct reproducible?
- Were replicates performed?
- Was an internal control used?
- Was inhibition assessed?

Even a brief statement such as “All reactions included internal controls to monitor inhibition…” would strengthen credibility.

The limitations section is appropriate but incomplete. In addition to small sample size and Ct interpretation ambiguity, please consider:

- Single-center study
- Postmortem-only population (selection bias toward severe cases)
- Lack of antigen testing
- No longitudinal clinical correlation
- No evaluation of treatment impact

Including at least two of these would strengthen credibility.

17) Minor language and style issues

Examples include:

- “need of integrating” → “need to integrate”
- “Live biopsies” → likely “Liver biopsies”
- “112 112 cells/µL” → duplication error
- “time-consuming” → add hyphen
- “such as molecular methods, such as qPCR” → avoid repetition

Final Evaluation

The manuscript requires **major revision**, as several points need clarification and further development.

- **Scientific relevance:** ★★★★★
- **Methodological robustness:** ★★★★☆
- **Interpretative caution:** ★★★☆☆
- **Clarity and conciseness:** ★★★☆☆
- **Impact potential after revision:** ★★★★★

With careful revision addressing the points above, the manuscript has strong potential for publication in a high-impact infectious diseases journal.
