# Supplementary material for: Tissue distribution of Histoplasma capsulatum in deceased people living with HIV assessed by minimally invasive tissue sampling: An autopsy-based case series in the Brazilian Amazon
Source: PLoS Negl Trop Dis. 2026 Aug 6;20(8):e0014545. doi: 10.1371/journal.pntd.0014545 (PMC13446691; doi:10.1371/journal.pntd.0014545)
Supplement: Supplementary file 2 [file pntd.0014545.s001.docx]

| **S1 Table:** Clinical and demographic characteristics at hospital admission of people living with HIV (PLHIV) included in the MITS study | | | | | | | | | | | | | |
| --- | --- | --- | --- | --- | --- | --- | --- | --- | --- | --- | --- | --- | --- |
| **ID** | **Age** | **Sex** | **HIV**  **load**  **(copies/mL)** | **Last**  **CD4**  **Count (cells/mm³)** | **Antiretroviral treatment** | **Time since HIV infection diagnosis (in months) to admission** | **Opportunistic diseases** | **Symptoms at admission** | **Clinical or imaging diagnosis of histoplasmosis** | **Antifungal**  **Treatment** | **Time between admission and death (days)** | **Cause of death** |  |
| 1 | 26 | M | >100.000 | <200 | Yes  (Dolutegravir + Lamivudine + Tenofovir) | 0.2 | Kaposi’s Sarcoma | Fever, weight loss, skin lesions, edema, respiratory symptoms, nausea, and vomiting. | No | No | 34 | Kaposi's Sarcoma |  |
| 3 | 19 | M | 90.262 | <200 | Yes  (Dolutegravir + Lamivudine + Tenofovir) | 130.5 | Pulmonary tuberculosis, esophageal candidiasis | Fever, skin lesions, respiratory symptoms. | No | No | 57 | Undetermined |  |
| 5 | 43 | M | 26.710 | 210 | No | 0 | Pulmonary tuberculosis and histoplasmosis | Fever, weight loss, lymphadenopathy, respiratory symptoms, focal neurological changes. | Yes | Conventional Amphotericin B, Fluconazole | 10 | Bacterial pneumonia |  |
| 7 | 39 | M | 262.287 | Nd | No | 0.8 | Histoplasmosis | Fever, weight loss, edema, oliguria, diarrhea | Yes | Conventional Amphotericin B, Fluconazol | 4 | Disseminated histoplasmosis |  |
| 9 | 40 | M | 293.595 | <200 | Yes  (Tenofovir + Lamivudine) | 124.9 | Histoplasmosis | Diarrhea, weight loss, oral mucosa injury | Yes | Conventional Amphotericin B Fluconazole (empirical treatment) | 25 | Undetermined |  |
| 10 | 25 | M | 1.800.000 | <200 | No | 11.3 | Pulmonary tuberculosis, Pneumocystosis | Fever, weight loss, respiratory symptoms, nausea, vomiting. | No | No | 1 | Undetermined |  |
| 11 | 27 | F | 398.159 | 229 | No | 281 | Oral candidiasis, hepatic  histoplasmosis and tuberculosis | Fever, weight loss, respiratory symptoms, vomiting. | No | No | 4 | Tuberculosis and  Pneumonia |  |
| 14 | 44 | M | 2.440.985 | <200 | Yes  (Dolutegravir + Lamivudine + Tenofovir) | 0.8 | Tuberculosis | Fever, weight loss, lymphadenopathy, respiratory symptoms. | No | No | 14 | Diffuse alveolar damage, |  |
| 15 | 42 | M | 1.063.937 | <200 | No | 2.6 | Histoplasmosis | Nausea, vomiting, diarrhea, fever. | Yes | Conventional Amphotericin B, Fluconazole (empirical treatment) | 7 | Diffuse alveolar damage |  |
| 26 | 35 | M | Nd | Nd | No | 1 | Toxoplasma infection | Fever, weight loss, focal neurological changes, respiratory symptoms, respiratory insufficiency | No | No | 33 | Intra-alveolar  hemorrhage |  |
| 34 | 40 | M | 317.154 | <200 | Yes | 0.5 | Pneumocystosis, cytomegalovirus and histoplasmosis | Fever, Weight loss, respiratory symptoms, respiratory insufficiency, diarrhea, nausea, and vomiting. | Yes | Conventional Amphotericin B | 14 | Pulmonary  histoplasmosis,  pulmonary  cytomegalovirus and  hepatic  histoplasmosis |  |
| 43 | 31 | M | 90 | <200 | Yes  (Tenofovir + Lamivudine) | 22 | Tuberculosis and Kaposi's sarcoma | Weight loss, oral mucosa injury, respiratory symptoms, nausea, vomiting | Yes | Fluconazole | 6 | Fungal lung disease  and aspiration  pneumopathy |  |
| 44 | 38 | M | Nd | Nd | No | 1 | Nd | Respiratory symptoms | No | No | 1 | Pneumopathy; Undetermined |  |
| 49 | 74 | M | Not detected | <200 | Yes  (Dolutegravir + Lamivudine + Tenofovir) | 56 | Nd | Fever, respiratory symptoms. | No | No | 2 | Undetermined |  |
| 50 | 46 | M | 178.903 | <200 | Yes  (Tenofovir + Lamivudine) | 78.4 | Cytomegalovirus | Fever, weight loss, respiratory symptoms, respiratory insufficiency, diarrhea, nausea, vomiting. | Yes | Conventional Amphotericin B, Itraconazole (empirical treatment) | 9 | Acute respiratory failure |  |
| 52 | 70 | M | 368.306 | <200 | Yes  (Dolutegravir + Lamivudine + Tenofovir) | 2.1 | Histoplasmosis | Fever, skin lesions, oral mucosa injury, diarrhea. | No | No | 70 | Pulmonary histoplasmosis, hepatic histoplasmosis |  |
| 53 | M | 32 | 2.982.869 | <200 | Yes  (Abacavir, Tenofovir + Lamivudine) | 1.2 | Kaposi's sarcoma, Cryptococcosis | Fever, weight loss, skin lesions, respiratory symptoms, nausea, vomiting. | No | No | 36 | Pulmonary cryptococcosis,  pulmonary Kaposi's sarcoma |  |
| 57 | 33 | M | Nd | <200 | Nd | 0.5 | Pneumocystosis | Fever, weight loss, respiratory symptoms, nausea, vomiting | No | No | 16 | Respiratory failure, bacterial pneumonia, pneumocystosis |  |
| 61 | 37 | M | 42.905 | <200 | Yes  (Tenofovir + Lamivudine) | 2 | Toxoplasma infection | Oral mucosa injury, respiratory symptoms, diarrhea, nausea, vomiting. | No | No | 15 | Undetermined |  |
| 62 | 48 | M | 96 | 280 | Yes  (ND) | 106 | Nd | Fever, weight loss, lymphadenopathy, respiratory symptoms, respiratory insufficiency, edema, diarrhea, nausea, vomiting | No | No | 1 | Undetermined, pneumopathy |  |
| 63 | 48 | F | 153.173 | 200 | Yes  (Zidovudine, Lamivudine, Efavirenz) | 138 | Tuberculosis, Cryptococcosis and Neurotoxoplasmosis | Weight loss, respiratory symptoms, respiratory insufficiency, diarrhea, hepatomegaly, intestinal bleeding, nausea, vomiting. | No | No | 24 | Cryptococcal  encephalitis and  bacterial pneumonia |  |
| 64 | 34 | M | Nd | <200 | No | 0.5 | Pneumocystosis | Fever, weight loss, oral mucosa injury, respiratory symptoms. | No | No | 15 | Pneumocystis |  |
| 65 | 53 | M | Nd | Nd | Yes  (Tenofovir + Lamivudine) | 0.6 | Nd | Weight loss, oliguria, respiratory symptoms, nausea, vomiting, diarrhea | No | No | 18 | Undetermined |  |
| 66 | 66 | F | Nd | <200 | No | 0.1 | Nd | Fever, weight loss, respiratory symptoms, diarrhea, nausea, vomiting. | No | No | 2 | Pneumonia |  |
| 68 | 34 | M | Nd | <200 | No | 3 | Cryptococcosis | Fever, weight loss, respiratory symptoms. | No | No | 2 | Disseminated cryptococcosis, |  |
| 69 | 42 | M | 108.613 | 280 | No | 53 | Tuberculosis | Weight loss, skin lesions, oliguria, respiratory symptoms, diarrhea, nausea, vomiting. | No | No | 3 | TB pulmonary and hepatic tuberculosis |  |
| 70 | 47 | F | Nd | Nd | Nd | 0.3 | Pneumonia, Cryptococcosis | Fever, skin lesions, genital mucosa injury, respiratory symptoms. | No | No | 7 | Histoplasmosis, Pulmonary cryptococcosis |  |

**Abbreviations:** PCR, polymerase chain reaction; qPCR, quantitative polymerase chain reaction; CSF, cerebrospinal fluid; Ct, cycle thre
