# Supplementary material for: Tissue distribution of Histoplasma capsulatum in deceased people living with HIV assessed by minimally invasive tissue sampling: An autopsy-based case series in the Brazilian Amazon
Source: PLoS Negl Trop Dis. 2026 Aug 6;20(8):e0014545. doi: 10.1371/journal.pntd.0014545 (PMC13446691; doi:10.1371/journal.pntd.0014545)
Supplement: Supplementary file 3 [file pntd.0014545.s002.docx]

| **Supplementary Table 2:** Characteristics at moment of HIV diagnosis | | | | |
| --- | --- | --- | --- | --- |
| **ID** | **HIV**  **load**  **(copies/mL)** | **Last**  **CD4**  **Count (cells/mm³)** | **Opportunistic diseases** | **Symptoms at diagnosis** |
| 1 | >100.000 | <200 | Kaposi's sarcoma | Asthenia, dyspnea, fever, dry cough, nausea, vomiting, weight loss and papules in the genital region |
| 3 | 90.262 | <200 | No | Cough |
| 5 | 26710 | 210 | Pulmonary tuberculosis | Progressive worsening of respiratory status, requiring intubation and hemodynamic instability, cold and cyanotic extremities, with thready radial pulses, cyanosis in both feet, and a generally harsh breath sound. Fever, jaundice (+/4+), and verrucous lesions in the genital region and lesions on the buccal mucosa. |
| 7 | Nd | Nd | Nd | ND |
| 9 | >100.000 | <200 | Nd | Odynophagia and Diarrhea |
| 10 | 1.800.000 | <200 | Pulmonary tuberculosis | Secretive cough, respiratory distress, high fever of 39°C, weight loss, lethargy and drowsiness, hypotension, weight loss, paleness, back pain, nausea and vomiting, skin spots all over the body, whitish lesions in the oropharynx and dehydration. |
| 11 | Nd | Nd | Nd | Nd |
| 14 | >100.000 | <200 | Bacterial pneumonia | Nd |
| 15 | >100.000 | <200 | Nd | Nd |
| 26 | Nd | Nd | Nd | Nd |
| 34 | >100.000 | <200 | Nd | Nd |
| 43 | Nd | ND | Nd | Nd |
| 44 | Nd | ND | Nd | Nd |
| 49 | Nd | 529 | Nd | Nd |
| 50 | >100.000 | <200 | Pulmonary Tuberculosis and Cytomegalovirus Esophagitis | Dyspnea., chest pain, retrosternal chest pain, unmeasured fever, productive cough with yellowish secretion, nausea and vomiting. |
| 53 | >100.000 | <200 | Nd | Nd |
| 57 | Nd | Nd | Nd | Nd |
| 61 | 42.905 | <200 | Nd | Nd |
| 62 | 7.823 | 463 | Nd | Nd |
| 63 | >100.000 | 200 | Nd | Nd |
| 64 | Nd | Nd | Nd | Nd |
| 65 | Nd | Nd | Nd | Nd |
| 66 | Nd | <200 | Nd | Nd |
| 68 | Nd | <200 | Nd | Nd |
| 69 | Nd | Nd | Nd | Nd |
| 70 | Nd | Nd | Nd | Nd |

Nd: Not done
