# Supplementary material for: Tissue distribution of Histoplasma capsulatum in deceased people living with HIV assessed by minimally invasive tissue sampling: An autopsy-based case series in the Brazilian Amazon
Source: PLoS Negl Trop Dis. 2026 Aug 6;20(8):e0014545. doi: 10.1371/journal.pntd.0014545 (PMC13446691; doi:10.1371/journal.pntd.0014545)
Supplement: Supplementary file 4 [file pntd.0014545.s005.docx]

**We thank the reviewers for their thorough analysis of our manuscript and for their valuable and constructive contributions.**

**We have carefully revised the text, addressing all observations, to improve clarity, increase methodological transparency, and strengthen the coherence between the study's objectives, results, and conclusions.**

**Below, we present a detailed, point-by-point response to each reviewer's comment, indicating the modifications implemented in the manuscript.**

Reviewer #1: The methods are clearly described. However, it is not clear, how the load of Histoplasma found in specific organs correlates to diagnostic accuracy. It depends on the clinical form of histoplasmosis. In pulmonary histoplasmosis, the lungs would probably have the highest loads, in disseminated forms it will depend on the organs which are infected. This can vary per patient. This is not addressed in the manuscript methods section.

**Answer: The aim of this study was not to evaluate the diagnostic accuracy of different tissues or methods, but rather to describe the distribution of fungal load in various organs obtained through MITS. We acknowledge that the fungal load observed in a specific tissue does not directly translate into improved diagnostic performance, as this relationship is strongly influenced by the clinical form of the disease and individual patient heterogeneity. Following the reviewer's suggestion, we have added further clarification in the Methods section and reinforced this limitation in the Discussion.**

line 163: this was postmortem material. Why could it have been limited? Why didn't the researchers take more material?

**Answer:**

**The MITS procedure, although following a standardized protocol with multiple punctures per organ, may present limitations in the amount of tissue obtained due to factors inherent to both the post-mortem context and the characteristics of the method. In this situation, tissue availability may be affected by the degree of preservation, including autolysis and tissue degradation processes. Furthermore, anatomical accessibility may hinder the collection of multiple representative fragments in certain regions. It is important to emphasize that MITS is a minimally invasive needle sampling technique and, therefore, does not replace a complete autopsy; its purpose is to balance adequate tissue representativeness with less invasiveness. Despite these limitations, whenever possible, multiple samples per organ were collected, generally between two and four punctures, as detailed in the Methods section.**

Reviewer #2: Objectives: the objective includes correlating fungal load, identifying optimal tissues, guiding clinical sampling, improving timeliness, and focusing on immunosuppressed individuals. This is overly broad. Please consider simplifying to Primary objective: Correlate fungal load with tissue distribution, and Secondary implication: Inform diagnostic strategies.
**Answer: The manuscript was revised according to the suggestions, and all points are addressed in detail below. In particular, the study objectives were simplified to clearly distinguish the main objective.**

Study design is apropriated but lacks specificityis (see the attached document).
Population cleary described.
Sample size is sufficient.
Essential corrections are need on the statistical analysis (see the attached document).
Ethics approval is properly mentioned with the CAAE number.

Reviewer #1: The results are not well described. Especially the results concerning the answers to the research question are only presented in a table (table 1) and are not stratified by clinical form of histoplasmosis. THis should be presented much more elaborated and stratified by clinical disease form (disseminated histo, pulmonary histo, extrapulm histo) not only in one table but also in the text.

**Answer: We have substantially revised the Results section to more precisely address the main research question. Instead of relying solely on Table 1, we now offer a detailed narrative description of the tissue distribution of *Histoplasma capsulatum* in the text.**

**To enhance clarity, we have introduced predefined tissue distribution categories based on postmortem findings: detection in multiple organs, detection restricted to the lungs, and detection in an isolated organ or body fluid. Considering that this is a descriptive autopsy-based study using MITS and not a clinical cohort, these categories were deemed more appropriate than conventional clinical classifications (such as disseminated, pulmonary, or extrapulmonary histoplasmosis), which depend on antemortem clinical presentation and diagnostic criteria not fully applicable postmortem. Thus, we have included a new subsection entitled "Tissue Distribution of *Histoplasma capsulatum*," in which the distribution patterns are described in detail.**

**We have also added Table 3, which summarizes organ involvement according to these predefined categories, allowing the reader to compare tissue distribution among the different infection patterns, rather than relying exclusively on the general frequencies presented in Table 1.**

Also, the added value of PCR in cases which were culture negative was not described. Were these true infections? Or were these only latent infections? And in these specific culture negative, PCR positive cases, which materials were PCR positive? What are the patient characteristics in these cases? Was histoplasmosis the cause of death or was there another cause of death and was Histoplasma found, but not of clinical relevance?
I miss the answers to the question: "How and when is MITS/PCR of added value?"
In table 2. I miss data on the diagnostics. Which tests of which organs were performed, and what were the results of culture and PCR of all materials of these patients. So add data from table 1 to table 2.

**Answer: The manuscript was substantially revised to better highlight the complementary contribution of PCR and qPCR, along with fungal culture and histopathology. Initially, we emphasized that the objective of the study was not to evaluate the diagnostic accuracy of PCR, but rather to characterize the anatomical distribution of Histoplasma capsulatum in post-mortem tissues using complementary diagnostic methods. Thus, we explicitly stated throughout the Methods and Discussion sections that molecular positivity should be interpreted as evidence of fungal DNA presence, not as proof of viable organisms or active infection.**

**To respond to the reviewer's observations, we expanded the Results section, including Table 4, which summarizes the results of culture, conventional PCR, qPCR, histopathology, and qPCR Ct values by organ. This table clearly shows the tissues in which molecular methods detected fungal DNA despite negative culture, demonstrating that PCR positivity occurred in multiple organs, notably the liver, lungs, blood, brain, and cerebrospinal fluid. We have also added a specific subsection detailing the contribution of molecular methods to tissue detection.**

**In addition, Table 2 has been revised to incorporate the individual results of all diagnostic methods performed on each patient and sampled organ, along with the predefined category of tissue distribution, the pathological assessment of histoplasmosis's role in death (primary, contributory, or unrelated), and the clinical interpretation of the cause of death. This organization allows readers to distinguish patients in whom *H. capsulatum* was considered clinically relevant from those in whom only fungal DNA was detected, without attributing histoplasmosis as the primary cause of death.**

**Regarding the possibility that positive PCR and negative culture results represent latent infection, we believe that our data do not support this conclusion. Since this is a descriptive post-mortem study, molecular detection was cautiously interpreted as evidence of fungal DNA. We now explicitly discuss how PCR positivity near the analytical limit of detection may reflect a low DNA load, residual DNA, or postmortem degradation. Therefore, these findings should not be seen as indicative of latent infection or viable organisms, but rather as complementary pathological information that, when integrated with culture, histopathology, tissue distribution, and overall pathological assessment, provides a more comprehensive characterization of tissue involvement.**

**The Discussion has been enhanced to better explain the added value of combining MITS with molecular methods. MITS enables standardized sampling of multiple organs in contexts where conventional autopsy is unavailable, while PCR increases sensitivity for detecting fungal DNA in tissues that remain culture-negative due to low fungal load, prior antifungal use, focal distribution within the tissue, or post-mortem changes. These complementary approaches contribute to a more accurate pathological characterization of disseminated histoplasmosis and can support mortality surveillance and the development of tissue-based diagnostic strategies in endemic settings.**

Lines 270-274: stratify these results according to clinical form (disseminated, lung. extrapulmonary). It makes no sense to present these data unstratified.

**Answer: Instead of limiting ourselves to general frequencies, we introduced predefined tissue distribution categories based on post-mortem findings: detection in multiple organs, detection restricted to the lung, and detection in a single organ or body fluid. The Results section now includes a subsection that describes these patterns in detail, and Table 3 summarizes organ involvement by category.**

**We chose not to stratify the findings by the conventional clinical forms of histoplasmosis (disseminated, pulmonary, and extrapulmonary), as our study is based exclusively on post-mortem samples obtained via MITS, without prior clinical evaluation. These clinical classifications depend on symptomatic presentation, imaging studies, microbiological investigations, and therapeutic evaluation, information that cannot be reliably reconstructed solely from post-mortem data.**

**Our objective was to characterize the anatomical distribution of *Histoplasma capsulatum* in tissues sampled by MITS. To avoid ambiguity, we explicitly clarify in the Methods and Discussion sections that these categories describe patterns of tissue involvement, not clinical diagnostic classifications. We believe this approach more accurately reflects the study's pathological nature while also fulfilling the reviewer's request for a stratified presentation of the results.**

Reviewer #2: The data presented in the Results section are clinically important and potentially impactful, particularly given the high prevalence of histoplasmosis in advanced HIV. However the Results section requires statistical clarification, table restructuring, and removal of overinterpretation. Essential corrections include:
• Replace all “median ± SD” with appropriate statistical format.
• Clarify denominators for all percentages.
• Remove unsupported sensitivity/specificity claims.
• Define qPCR positivity threshold.
• Reformat Table 1 with a clear legend and standardized notation.
• Clearly explain classification criteria.

**Answer: The textual changes follow.**

**• Statistical format: Continuous variables previously reported as “median ± SD” have been corrected and are now presented as median (interquartile range), in line with standard statistical reporting practices.**

**• Denominators: Percentages throughout the Results section have been revised to include explicit denominators (n/N), enhancing clarity and transparency.**

**• Sensitivity/specificity: Statements suggesting diagnostic sensitivity or specificity have been removed or rephrased, since the study design does not permit formal assessment of these parameters. The Results now emphasize descriptive comparisons between methods.**

**• qPCR threshold: The qPCR positivity threshold (Ct≥ 38) is clearly defined in the Methods and consistently applied and referenced in the Results.**

**• Table 1: The table has been reformatted to improve clarity with standardized notation (Positive/Negative), a clearer structure, and an expanded legend detailing diagnostic methods and definitions.**

**• Classification criteria: Criteria for categorizing histoplasmosis as primary cause of death, contributory factor, or not clinically relevant are explicitly defined in the Methods section and referenced in the Results.**

Reviewer #1: The conclusion is not supported by the data presented.
The limitations of the study are not clearly described.
**Answer: We substantially revised the Discussion, Limitations, and Conclusion sections to ensure that all interpretations are rigorously grounded in the results presented. The Conclusion was completely reformulated to more accurately reflect the study's descriptive nature. Statements that could suggest diagnostic superiority of molecular methods or unduly broaden clinical applicability beyond the scope of the data were removed. The new wording emphasizes that the main finding is the characterization of the post-mortem tissue distribution of *Histoplasma capsulatum* in individuals with HIV, using MITS combined with complementary microbiological, histopathological, and molecular techniques. In addition, we explicitly highlight that the results should not be directly extrapolated to clinical practice, emphasizing the need for prospective studies to validate diagnostic strategies based on tissue samples.**

**The Limitations section has been significantly expanded to include more detailed aspects such as the relatively small sample size, the single-center design, the exclusivity of deceased patients with advanced HIV, the selection bias associated with case severity, the post-mortem investigation, the possible effects of autolysis and DNA degradation, the variability in the amount of tissue obtained by MITS depending on anatomical accessibility, the possible influence of previous antifungal treatments on microbiological findings, and the absence of an independent gold standard for evaluating diagnostic accuracy.**

In the discussion, several statements are made that are much broader than what can be concluded from the results of this study. These statements have not been investigated, and no sources are provided. As a result, it almost becomes a kind of 'expert opinion,' which is not the purpose of this study.
**Answer: We thank the reviewer for this important observation. In response, we have extensively revised the Discussion section to ensure that all interpretations remain strictly aligned with the data presented and properly contextualized by the available literature.**

Line 331-332: we miss nuance on the meaning of a positive PCR. Is this 25,9% of the 18% with diagnosis histoplasmosis or 25,9% of the whole group. Please clarify, and also add this to the manuscript.

**Answer: The percentage (25.9%) refers to patients with confirmed histoplasmosis (7/27), rather than the total study population.**

Line 361: rapid diagnosis: but then the clinicians should think about histoplasmosis first. Before you write, that this is a major problem in diagnostics.

**Answer: In response, we revised the Discussion section to reflect this perspective in a more balanced way. The text now highlights that the timely diagnosis of histoplasmosis depends on both clinical suspicion and access to appropriate diagnostic methods, including antigen detection and molecular tests. In this way, we avoid attributing diagnostic delays solely to laboratory limitations and emphasize that both factors contribute to the underdiagnosis of the disease in endemic regions.**

Lines 363-368: the authors should be able to show resilts of this? They should have this information? I think this information is essential to describe, before you can conclude that PCR is superior to detect infections.

**Answer: In response, we substantially revised the manuscript to avoid this interpretation. We removed statements suggesting the superiority of PCR over culture or histopathology and emphasized that this study was not designed to evaluate diagnostic accuracy or compare the performance of different laboratory methods. We also clarified that a positive PCR result represents the detection of Histoplasma capsulatum DNA and should not be interpreted, in isolation, as evidence of viable organisms or active infection.**

Line 380: this cannot be concluded. This is dependent of the localization of the infection and cannot be generalized like this.

**Answer: In response, we revised this section of the Discussion to avoid generalized recommendations about the best sites for tissue sampling. The revised text makes it clear that our results reflect the anatomical distribution observed in a post-mortem series of patients with advanced HIV and should not be directly extrapolated to clinical practice.**

**We now emphasize that the predominance of Histoplasma capsulatum detection in the liver and lungs represents a histopathological finding and that the choice of tissue for diagnostic investigation in living patients depends on the clinical context, the presentation of the disease, the accessibility of the organ, and the suspicion of specific involvement. Thus, we reformulated the discussion to indicate that these findings may guide hypotheses and future prospective studies, but do not constitute clinical recommendations.**

Lines 395-397: this is not investigated in this study and no reference is added. So, please add a reference. Otherwise remove this section.

**Answer: We agree with the reviewer and have added an appropriate reference to support this statement.**

Lines 401-402: this is not investigated in this study and no reference is added. So, please add a reference. Otherwise remove this section.

**Answer:** **We agree with the reviewer and have added an appropriate reference to support this statement.**

Use this section for editorial suggestions as well as relatively minor modifications of existing data that would enhance clarity. If the only modifications needed are minor and/or editorial, you may wish to recommend “Minor Revision” or “Accept”.

Reviewer #1: Minor comments: Throughout the manuscript 0, 1 or 2 decimals are used in the Ct values. Please use 1 decimal for Ct values throughout the manuscript.
line 105-107: grammar not OK, please rephrase
line 137: change 2,063,547 into approximately 2 million
Line 156: change colected into collected
Table 1: Wat does LCR mean?
Table 1: what do the numbers 43, 44, 28, 22 and so on mean? I suggest Ct values. Please clarrify and add to the text.

Line 271: what do the authors mean with 81TT.4%?
Line 311: change people living with HIV into PLHIV
Line 331: change them into they
Line 349: write Histoplasma in italics

**Answer:**

**• We standardized all Ct values to one decimal place throughout the manuscript and tables.**

**• The text in lines 105–107 was rewritten to improve grammar and clarity.**

**• The value 2,063,547 was replaced with approximately 2 million, as suggested.**

**• The typo "colected" was corrected to "collected".**

**• The abbreviation LCR was replaced with CSF (cerebrospinal fluid), and its meaning was clarified in the table and text.**

**• The numerical values presented in Table 1 (e.g., 43, 44, 28, and 22) were explicitly identified as qPCR Ct values, and this information was added to both the table header/legend and the Methods section.**

**• The typographical error "81TT.4%" was corrected to 81.4%.**

**• The expression "people living with HIV" has been replaced with "PLHIV," in accordance with the standardization adopted throughout the rest of the manuscript.**

**• The pronoun "them" has been corrected to "they," as appropriate to the context of the sentence.**

**• The genus name *Histoplasma* has been standardized in italics throughout the manuscript.**

Reviewer #2: The manuscript needs major revision. See suggestions ont the attached document.

Reviewer #1: The study describes an interesting technique for the diagnosis of histoplasmosis in HIV postive patients. The methods are clearly described. However the results are not well described and do not support the conclusions made. The conclusions in the discussion cannot be drawn from this study.
Abstract: Conclusion too strong
Author summary: Conclusion too strong and specify what is the gold standaard?

**Answer:** **In response, we conducted a comprehensive review of the manuscript. The Results section was reorganized and expanded to present a more detailed account of the tissue distribution of *Histoplasma capsulatum*, including a narrative description of the findings and new tables that stratify the patterns of tissue involvement and present, individually, the results of culture, histopathology, conventional PCR, and qPCR for each patient and organ analyzed.**

**The Discussion was substantially revised to ensure that all interpretations remained strictly based on the data obtained. We removed statements that could be interpreted as extrapolations or as demonstrating the diagnostic superiority of PCR, adopted more cautious language, and included additional references to support interpretations based on the literature. We also emphasized that this study is descriptive in nature and was not designed to assess diagnostic accuracy or compare the performance between methods.**

**The Conclusion of the manuscript, as well as the Abstract and Author Summary, was rewritten to more adequately reflect the scope of the study. The conclusions now emphasize that the main finding is the characterization of the post-mortem tissue distribution of H. capsulatum, achieved using MITS integrated with complementary microbiological, histopathological, and molecular methods, while avoiding extrapolations to clinical practice. In the Author Summary, we also clarify that fungal culture is traditionally considered the reference standard, although it has recognized limitations, particularly in disseminated histoplasmosis.**

**We have significantly expanded the Limitations section, discussing in more detail the restrictions inherent in the study design, including sample size, single-center nature, exclusive inclusion of deceased patients, limitations of MITS, the absence of an independent gold standard for evaluating diagnostic performance, and the need for prospective studies to validate the findings.**

**Revisor 2**

**1) Introduction section**

There is some conceptual redundancy, minor inaccuracies or overgeneralizations, and overstatement in certain claims. The study rationale also requires sharper refinement. For instance, in the study aim paragraph (Page 10, Line 126), the authors state: “identifying which tissues have the most significant potential for clinical sampling among alive patients…”

**Answer: We revised the entire Introduction to reduce conceptual redundancies, eliminate overgeneralizations, and make the study's rationale more objective and aligned with its actual goals.**

**Specifically, we reformulated the study's objective to emphasize that our purpose was to characterize the post-mortem tissue distribution of Histoplasma capsulatum using MITS integrated with microbiological, histopathological, and molecular methods, and not to identify the best biopsy sites or establish recommendations for living patients.**

**In addition, we revised the language throughout the Introduction to avoid overly broad statements and restricted potential clinical implications to the Discussion, where they are cautiously presented as hypotheses for future prospective investigations, and not as conclusions of the present study.**

Additionally, the objective includes correlating fungal load, identifying optimal tissues, guiding clinical sampling, improving timeliness, and focusing on immunosuppressed individuals. This is overly broad. Please consider simplifying to:

 Primary objective: Correlate fungal load with tissue distribution.

 Secondary implication: Inform diagnostic strategies.

High-impact journals generally prefer focused objectives.

**Answer: We agreed that the original version of the objective was overly broad and included potential clinical implications that went beyond the scope of the present study.**

**In response, we completely reformulated the objective to make it more specific and aligned with the research design. The revised objective focuses on characterizing the post-mortem tissue distribution of Histoplasma capsulatum using minimally invasive tissue sampling (MITS) integrated with microbiological, histopathological, and molecular methods, as well as describing the distribution of fungal load among different tissues.**

**The possible implications for diagnostic strategies and future clinical applications were removed from the objective and are now discussed exclusively in the Discussion section, where they are presented as potential developments of the findings, and not as objectives or conclusions of the study.**

The flow between paragraphs could also be smoother, and language tightening is

required in several places, such as:

 Page 9, Line 101: Histoplasma should be italicized.

 Page 9, Line 102: “through public health systems” → change to “within public

health systems.”

 Page 10, Line 125: “to correlate histoplasma load” → capitalize and italicize

Histoplasma.

 Page 10, Line 127: “among alive patients” → change to “among living patients.”

 Some sentences exceed 35–40 words; consider splitting them for clarity.

**Answer: In response, we carefully revised this entire section, improving the transition between paragraphs, reducing redundancies, and making the justification for the study more objective and focused. Furthermore, we incorporated all the specific corrections suggested by the reviewer.**

**2) Study design lacks specificity**

Page 10, Line 133: “This study is observational in a series of cases” is grammatically

imprecise.

The appropriate terminology would be:

 “observational case series study” or  “observational autopsy-based case series.”

**Answer: The sentence has been revised accordingly, and the study design is now described as an “observational autopsy-based case series.”**

**3) Epidemiological justification could be better connected**

Page 11, Lines 138–140: The 34% finding is relevant, but a sentence explicitly linking

this result to the need for the present study is missing.

**Answer: We agree that the original version of the Introduction did not sufficiently explicitly establish the connection between the high frequency of histoplasmosis observed in previous studies and the rationale for the present study.**

**In response, we revised this passage to highlight that, although previous studies have demonstrated the high burden of histoplasmosis among people living with HIV in the Amazon, there are still important gaps related to the anatomical distribution of *Histoplasma capsulatum* in different tissues and the contribution of microbiological, histopathological, and molecular methods to its post-mortem characterization.**

**Thus, we added a transition sentence explaining that this knowledge gap motivated the present study, which aims to characterize the tissue distribution of the fungus using MITS integrated with complementary diagnostic methods.**

**4) Incorrect geographic location**

Page 11, Line 136: Manaus is located in the Northern Region of Brazil, not in the

“Northeast of the Amazonian State.” This should be corrected to avoid a geographic

error.

**Answer: We revised the text to correctly indicate that Manaus is located in the state of Amazonas, in the Northern Region of Brazil.**

**5) Exclusion criteria not described**

Please clarify whether cases with advanced decomposition, known prior coinfections, or

marked autolysis were excluded.

**Answer: We explain the exclusion criteria more explicitly in the Methods section.**

**6) HIV diagnosis not defined**

It would be important to specify whether HIV infection was confirmed by laboratory

criteria or based solely on clinical records.

**Answer: We clarify in the Methods section that the diagnosis of HIV infection was established based on previously documented clinical records, which are supported by laboratory criteria consistent with current national guidelines.**

7) “Randomly punctured the tissues” This wording may raise methodological concerns. Random sampling could reduce diagnostic representativeness. Ideally, the manuscript should indicate: • Standardized number of punctures per organ • Approximate depth of sampling • Whether systematic anatomical orientation was followed

**Answer: We thank the reviewer for this important comment. We acknowledge that the original wording might have caused concerns about sampling representativeness. To address this, the manuscript has been revised to clarify that tissue collection followed a standardized MITS protocol. Specifically, we have added methodological details to enhance clarity and reproducibility:**

**• A standardized number of punctures per organ was performed, typically 2 to 4, whenever feasible to improve sample representativeness.**

**• Needle insertion depth was adjusted to ensure adequate access to the target organ parenchyma.**

**• All punctures were guided by systematic anatomical orientation, using predefined landmarks for each organ.**

**These clarifications confirm that sampling was standardized and anatomically directed rather than random.**

8) Reproducibility

To increase reproducibility, please include information about:

 Number of fragments collected per organ

 Minimum volume obtained

 Standardization across cases

**Answer: Specifically, we clarified that:**

**• A standardized number of tissue fragments was collected per organ, typically two to four punctures per organ whenever feasible.**

**• For liquid samples, a minimum volume of approximately 200 µL was obtained when possible; for solid tissues, fragments of around 25–50 mg were collected.**

**• The procedure adhered to a standardized protocol applied consistently across cases, with deviations occurring only due to tissue condition or sample availability constraints.**

9) Cross-contamination control

Given that molecular and mycological analyses were performed, it is important to

mention:

 Needle replacement between organs

 Anti-contamination measures

 Use of negative controls

**Answer: In response, we expanded the description of contamination control procedures in the Methods section to include the performance of molecular and mycological analyses.**

10) Molecular transport conditions

Page 12, Line 160: “Kept on cold chain” is vague. Please specify the exact temperature

(later mentioned as 2–8 °C) and the maximum time until processing.

**Answer: We specify that specimens intended for molecular analyses were transported and kept refrigerated between 2–8 °C, in a continuous cold chain, until laboratory processing. We also added the maximum time between collection and processing (or, when applicable, between collection and storage), making the methodological description more complete and reproducible.**

11) Clinical Information section

The section does not specify which demographic, epidemiological, or clinical variables

were extracted. For methodological transparency and reproducibility, please clarify

whether the following were collected:

• Age and sex

• HIV-related parameters (CD4 count, viral load, ART use)

• Opportunistic infections

• Prior antifungal therapy

• Comorbidities

• Laboratory parameters (e.g., liver enzymes, hematologic profile)

**Answer: We expanded the Clinical Information section of the Methods to explicitly specify which variables were extracted from medical and hospital records. The revised description includes demographic variables (age and sex), parameters related to HIV infection (including use of antiretroviral therapy and, when available, CD4+ lymphocyte count and viral load), presence of opportunistic infections and other relevant clinical conditions, prior use of antifungal therapy, as well as the main laboratory data available for clinical characterization of the patients.**

**We also clarified in the manuscript that some variables were limited in availability because the study was retrospective and based on post-mortem investigations, which is why not all were available for all patients.**

The section also does not specify how HIV diagnosis was confirmed (laboratory confirmation vs. clinical record), which is particularly relevant in autopsy-based studies.

**Answer: We revised the Clinical information / Patients included section to clarify that the HIV diagnosis was obtained from documented clinical records, based on laboratory confirmation according to standard care procedures.**

The term “epidemiological information” is broad and undefined. It would be helpful to specify whether place of residence (urban vs. rural), occupational exposure, travel history, or environmental exposure risks were collected.

**Answer: We revised the Methods section to specify exactly what epidemiological information was extracted from medical records and hospital registers. We also removed the generic use of the term, replacing it with an explicit description of the variables actually collected.**

**We further clarified that this retrospective study, based on post-mortem investigation, did not include systematic collection of detailed information on occupational exposure, travel history, or environmental risk factors, since this data was not consistently available in the clinical records analyzed. This limitation was acknowledged in the revised manuscript.**

Additionally, there is no mention of standardized data collection or double review

procedures. Please clarify:

 Whether data extraction followed a standardized case report form

 Whether more than one reviewer performed data abstraction

 How discrepancies were resolved

 Whether missing data were handled systematically

**Answer: In response, we have expanded the Methods section to clarify the process of collecting clinical and epidemiological data. We inform you that data extraction was performed using a standardized information collection form developed specifically for this study, ensuring uniformity in obtaining the variables of interest.**

**We also clarify that the data abstraction was performed by one researcher and subsequently reviewed by the other authors during the data consistency verification and results analysis stage. Any inconsistencies identified were resolved by consensus, through consultation of the original medical records.**

**In addition, we add that missing data were treated as missing data and were not subjected to imputation procedures, with analyses performed only with the information actually available for each variable.**

Given that retrospective record review is subject to information bias, this should be

addressed.

**Answer: In response, we included this limitation in the Discussion section, highlighting that the quality and completeness of the data depend on the available clinical records, which may result in underreporting or variability in the information collected.**

Overall, the section is clear but overly concise and methodologically underdeveloped.

Expanding it would significantly improve scientific rigor and reproducibility.

**Answer: We have substantially revised and expanded this section with the aim of increasing the methodological transparency, scientific rigor, and reproducibility of the study. Additional information has been included regarding the study design, case inclusion criteria, sample collection and processing procedures, transport and storage conditions, laboratory methods used, interpretation of molecular results, clinical and epidemiological variables extracted from medical records, data collection and verification process, and the handling of missing data.**

**We have also clarified methodological aspects that were not sufficiently detailed in the original version, including the rationale for the classification of tissue distribution patterns, the complementary role of the different diagnostic methods employed, and the limitations inherent in the observational design based on post-mortem investigation.**

12) Mycological analysis

It is unclear whether fungal identification was based solely on morphology and thermal

dimorphism testing or whether molecular confirmation was performed. Additionally, to

strengthen methodological robustness, please describe:

 Time to growth considered positive

 Whether repeat cultures were performed

 Biosafety level for Histoplasma handling (BSL-2 or BSL-3)

**Answer: We expanded the Methods section to clarify that the identification of *Histoplasma capsulatum* by culture was based on its macro and micromorphological characteristics, associated with the demonstration of thermal dimorphism, according to the laboratory's routine procedures. We also clarified the role of molecular methods in the study, highlighting that these were used as complementary methods for detecting fungal DNA in tissues and not as a tool for confirming the identification of isolates obtained in culture.**

**In addition, we added information about the microbiological protocol, including the maximum incubation time adopted to consider a culture negative, the performance (or not) of subcultures/repetition of cultures when necessary, and the level of biosafety used for handling *H. capsulatum*, according to current institutional standards and laboratory recommendations.**

13) Molecular methods – analytical performance

The manuscript does not report:

 Limit of detection (LOD)

 Analytical sensitivity and specificity

 Amplification efficiency

 Validation in autopsy specimens (if different from the original validation study)

**Answer: The qPCR assay used in this study was previously developed and submitted to analytical validation by Silva et al. (21), in which the limit of detection (LOD), analytical sensitivity and specificity, as well as amplification efficiency were determined and described in detail.**

**In response to the comment, we revised the Methods section to explicitly cite this validation study, thereby allowing the reader to directly access the assay's analytical characteristics. We also clarified that the objective of the present study was not to perform a new analytical validation or to compare the method's diagnostic performance, but to apply a previously validated assay to investigate the tissue distribution of *Histoplasma capsulatum* in samples obtained by MITS.**

Including these parameters would strengthen confidence in diagnostic performance.

**Answer: We agree that describing the analytical parameters can increase confidence in the interpretation of molecular results.**

**However, we chose not to reproduce these parameters in detail in this manuscript, since the qPCR assay used has already been developed and submitted to analytical validation by Silva et al. (21), where the limit of detection , analytical sensitivity and specificity, and amplification efficiency are presented. Instead, we revised the Methods section to explicitly cite this validation study, allowing the reader to access all the analytical characteristics of the assay.**

**We also clarified that the objective of this study was not to evaluate the diagnostic performance or revalidate the molecular method, but to apply a previously validated assay to characterize the tissue distribution of *Histoplasma capsulatum* in samples obtained by MITS. Additionally, we acknowledge in the Discussion that the assay has not yet been specifically validated for post-mortem samples derived from MITS, which we have incorporated as a limitation.**

Please also mention potential cross-reactivity with phylogenetically related fungi and

clarify how Ct values were interpreted.

**Answer: We expanded the description of the molecular methods and the Discussion to clarify both aspects.**

**First, we added that the qPCR assay used was previously developed and validated by Silva et al. (21), a study in which its analytical specificity against other microorganisms, including phylogenetically related fungi, was also evaluated. Thus, the potential for cross-reactivity was considered during the original method validation process.**

**In addition, we clarified in more detail the interpretation of Ct values. We specified that samples with Ct values close to the analytical detection limit were interpreted with caution, as they may reflect low fungal DNA load or DNA degradation due to the post-mortem process. Thus, we reinforced that qPCR positivity was considered as evidence of the presence of *Histoplasma capsulatum* DNA and not as a demonstration of viable organisms or active infection. Molecular results were always interpreted in conjunction with culture, histopathology, tissue distribution, and the anatomopathological context.**

14) Histopathological criteria

The morphological criteria used to define Histoplasma spp. in tissue are not described

(e.g., yeast size, intracellular location, narrow-based budding). Explicit criteria would

enhance reproducibility.

**Answer: We revised the Histopathological Methods section to clarify the diagnostic criteria applied. Specifically, we included a description of the classical morphological characteristics of *Histoplasma capsulatum* in tissue samples.**

15) Results section

The Results section requires statistical clarification, table restructuring, and removal of

overinterpretation. Essential corrections include:

 Replace all “median ± SD” with appropriate statistical format.

 Clarify denominators for all percentages.

 Remove unsupported sensitivity/specificity claims.

 Define qPCR positivity threshold.

 Reformat Table 1 with a clear legend and standardized notation.

 Clearly explain classification criteria.

**Answer: We revised the presentation of continuous variables, using the appropriate statistical notation for each data type and correcting the inadequate presentation of measures of central tendency and dispersion.**

**• We explicitly specified the denominators of all percentages presented in the text and tables, including their respective absolute values, avoiding ambiguities in the interpretation of the results.**

**• We removed statements that could be interpreted as an assessment of the sensitivity, specificity, or diagnostic superiority of molecular methods, making it clear that this study was not designed to compare diagnostic performance.**

**• We clarified the criteria used for the interpretation of qPCR, including the definition adopted for positivity and the cautious interpretation of Ct values close to the analytical limit of detection.**

**• We restructured Table 1, revising its legend, standardizing the terminology, abbreviations, and presentation of results to facilitate its understanding.**

**• We also expanded the description of the criteria used to classify tissue distribution patterns (multi-organ detection, lung-restricted detection, and single-organ/body-fluid detection), clearly explaining these concepts in both the Methods and Results sections.**

**In addition, we reorganized the Results section, incorporating a more detailed narrative description of the main findings and new tables that more clearly present the tissue distribution and the results of the different diagnostic methods.**

16) Discussion section

Lines 333–356 are conceptually strong but repetitive. The authors repeat:

 Nonspecific symptoms

 Overlap with tuberculosis

 Limited diagnostics in Latin America

 Regional lack of access

**Answer: We reviewed and reorganized these paragraphs to make the discussion more concise and fluid. Information on the nonspecific clinical presentation of histoplasmosis, its overlap with tuberculosis, limitations in access to diagnostic methods, and the epidemiological context of Latin America were integrated into a single block of text, eliminating repetitions without compromising the contextualization of the findings.**

**In addition, we took advantage of this review to broaden the international perspective of the discussion, including a brief contextualization of the underreporting and diagnostic difficulties of histoplasmosis in other endemic regions, such as Sub-Saharan Africa and Southeast Asia. In this way, the discussion became more objective, better structured, and more aligned with the scope of the study, preserving essential information and reducing redundancies.**

Please condense this into one cohesive paragraph emphasizing clinical overlap,

structural diagnostic limitations, and the consequences of delayed or missed treatment.

**Answer: We revised the text and condensed the content into a single, more cohesive paragraph, integrating these elements in a clearer and more objective way, emphasizing the clinical overlap with other endemic infections, the structural limitations of access to diagnosis in Latin America, and the consequences of delayed or failed diagnosis, including the impact on clinical outcomes.**

The interpretation of qPCR performance is the most critical methodological section.

The statement “qPCR performed remarkably well... demonstrating superiority...”

requires moderation because:

 The study uses postmortem tissue (MITS).

 There is no gold-standard comparator for all cases.

 Molecular detection does not necessarily indicate viable infection.

 Ct≥ 38 ambiguity is acknowledged but not fully explored.

**Answer: We revised the manuscript to moderate the previously used language (e.g., replacing “demonstrating superiority” with more appropriate terms, such as “demonstrated higher analytical sensitivity compared with culture”), avoiding over-interpretation. Furthermore, we more explicitly incorporated the following limitations: The use of post-mortem samples (MITS), which can influence molecular detection; The absence of a uniform gold standard for all cases; The fact that DNA detection does not necessarily indicate viable or active infection; and The cautious interpretation of elevated Ct values (especially≥ 38), which may reflect low fungal load, DNA degradation, or residual detection.**

To strengthen scientific rigor, please explicitly address:

 Risk of DNA persistence postmortem

 Risk of environmental contamination

 Lack of standardized Ct cut-offs

 Absence of antigen testing in most cases

**Answer: We condensed this section of the Discussion into a single, more cohesive and objective paragraph.**

**In the revised version, we integrated the discussion on the nonspecific clinical presentation of histoplasmosis, its overlap with tuberculosis and other opportunistic infections, the structural limitations of access to diagnostic methods in endemic regions, and the consequences of late or missed diagnosis. We also highlighted that diagnostic delay results not only from the limited availability of diagnostic tools, but also from low clinical suspicion, factors that together contribute to the late initiation of antifungal treatment and the high mortality associated with disseminated histoplasmosis.**

Instead of “superiority,” consider “demonstrated higher analytical sensitivity compared

with culture...”

**Answer: We revised the manuscript to replace that expression with a more appropriate and technically accurate formulation.**

Regarding Ct interpretation, please clarify:

 Was Ct reproducible?

 Were replicates performed?

 Was an internal control used?

 Was inhibition assessed?

**Answer: The qPCR assay employed in this study was performed according to the protocol previously developed and validated by Silva et al. (21), in which aspects related to assay reproducibility, use of internal controls, and analytical performance are described in detail. In this manuscript, we explicitly add this reference to direct the reader to the analytical characteristics of the method.**

**In addition, we clarify that Ct values were interpreted cautiously, especially when close to the analytical detection limit. In these cases, the results were considered evidence of the presence of Histoplasma capsulatum DNA and always interpreted in conjunction with microbiological, histopathological, and anatomopathological findings, not being used in isolation to infer active infection or viable organisms.**

**We also specify in the manuscript the controls used during the qPCR reactions and clarify the procedures adopted to ensure the analytical quality of the assay, as described in the original validation protocol.**

Even a brief statement such as “All reactions included internal controls to monitor

inhibition...” would strengthen credibility.

**Answer: We have added a brief description in the Methods section specifying the use of internal controls during qPCR reactions, clarifying their role in monitoring amplification quality and identifying possible reaction inhibition. We also emphasize that the interpretation of molecular results was carried out in conjunction with microbiological, histopathological, and anatomopathological findings.**

The limitations section is appropriate but incomplete. In addition to small sample size and Ct interpretation ambiguity, please consider:

 Single-center study

 Postmortem-only population (selection bias toward severe cases)

 Lack of antigen testing

 No longitudinal clinical correlation

 No evaluation of treatment impact

Including at least two of these would strengthen credibility.

**Answer: In response, we expanded the Limitations section to include additional**

17) Minor language and style issues

Examples include:

 “need of integrating” → “need to integrate”

 “Live biopsies” → likely “Liver biopsies”

 “112 112 cells/μL” → duplication error

 “time-consuming” → add hyphen

 “such as molecular methods, such as qPCR” → avoid repetition

**Answer: Changed**
