# Supplementary material for: Tissue distribution of Histoplasma capsulatum in deceased people living with HIV assessed by minimally invasive tissue sampling: An autopsy-based case series in the Brazilian Amazon
Source: PLoS Negl Trop Dis. 2026 Aug 6;20(8):e0014545. doi: 10.1371/journal.pntd.0014545 (PMC13446691; doi:10.1371/journal.pntd.0014545)
Supplement: Peer review history

# Peer review: Decision Letter 0

**DOI:** 10.1371/journal.pntd.0014545.r001

**Authors:** Marcio Rodrigues

3 Mar 2026

PNTD-D-25-01973

Parasitic burden of Histoplasma capsulatum in different tissues collected through minimally invasive tissue sampling in deceased patients with HIV/AIDS from the Brazilian Amazon

PLOS Neglected Tropical Diseases

Dear Dr. Baia,

Thank you for submitting your manuscript to PLOS Neglected Tropical Diseases. After careful consideration, we feel that it has merit but does not fully meet PLOS Neglected Tropical Diseases's publication criteria as it currently stands. Therefore, we invite you to submit a revised version of the manuscript that addresses the points raised during the review process.

Please submit your revised manuscript within by May 02 2026 11:59PM. If you will need more time than this to complete your revisions, please reply to this message or contact the journal office at plosntds@plos.org. When you're ready to submit your revision, log on to https://www.editorialmanager.com/pntd/ and select the 'Submissions Needing Revision' folder to locate your manuscript file.

Please include the following items when submitting your revised manuscript:

* A letter that responds to each point raised by the editor and reviewer(s). You should upload this letter as a separate file labeled 'Response to Reviewers'. This file does not need to include responses to any formatting updates and technical items listed in the 'Journal Requirements' section below.

* A marked-up copy of your manuscript that highlights changes made to the original version. You should upload this as a separate file labeled 'Revised Manuscript with Track Changes'.

* An unmarked version of your revised paper without tracked changes. You should upload this as a separate file labeled 'Manuscript'.

If you would like to make changes to your financial disclosure, competing interests statement, or data availability statement, please make these updates within the submission form at the time of resubmission. Guidelines for resubmitting your figure files are available below the reviewer comments at the end of this letter.

We look forward to receiving your revised manuscript.

Kind regards,

Marcio L Rodrigues

Section Editor

PLOS Neglected Tropical Diseases

Marcio Rodrigues

Section Editor

PLOS Neglected Tropical Diseases

Shaden Kamhawi

co-Editor-in-Chief

PLOS Neglected Tropical Diseases

orcid.org/0000-0003-4304-636XX

Paul Brindley

co-Editor-in-Chief

PLOS Neglected Tropical Diseases

orcid.org/0000-0003-1765-0002

**Journal Requirements:**

1) Please ensure that the CRediT author contributions listed for every co-author are completed accurately and in full.

At this stage, the following Authors/Authors require contributions: Karolina Jeaneth Solórzano-Chavarría, Taygo Silva Graciliano, Izabella Picinin Safe Lacerda, Alexandre Vilhena Silva-Neto, Luiz Ferreira, Monique Freire Santana, Katia Cruz, Carla Silvana Silva Santos, Juan Diego Ribeiro Almeida, Luciana Aires Oliveira, Jaume Ordi, Natalia Rakislova, Mikel Martinez, Juan Carlos Hurtado, João Vicente Braga Souza, Quique Bassat, Marcus Vinícius Guimarães Lacerda, and Djane Baia. Please ensure that the full contributions of each author are acknowledged in the "Add/Edit/Remove Authors" section of our submission form.

The list of CRediT author contributions may be found here: https://journals.plos.org/plosntds/s/authorship#loc-author-contributions

2) Please upload all main figures as separate Figure files in .tif or .eps format. For more information about how to convert and format your figure files please see our guidelines:

https://journals.plos.org/plosntds/s/figures

3) We notice that your supplementary Tables are included in the manuscript file. Please remove them and upload them with the file type 'Supporting Information'. Please ensure that each Supporting Information file has a legend listed in the manuscript after the references list.

4) Please amend your detailed Financial Disclosure statement. This is published with the article. It must therefore be completed in full sentences and contain the exact wording you wish to be published.

- State the initials, alongside each funding source, of each author to receive each grant. For example: "This work was supported by the National Institutes of Health (####### to AM; ###### to CJ) and the National Science Foundation (###### to AM)."

- State what role the funders took in the study. If the funders had no role in your study, please state: "The funders had no role in study design, data collection and analysis, decision to publish, or preparation of the manuscript.".

If you did not receive any funding for this study, please simply state: u201cThe authors received no specific funding for this work.u201d

**Reviewers' Comments:**

Reviewer's Responses to Questions

**Key Review Criteria Required for Acceptance?**

As you describe the new analyses required for acceptance, please consider the following:

**Methods**

-Are the objectives of the study clearly articulated with a clear testable hypothesis stated?

-Is the study design appropriate to address the stated objectives?

-Is the population clearly described and appropriate for the hypothesis being tested?

-Is the sample size sufficient to ensure adequate power to address the hypothesis being tested?

-Were correct statistical analysis used to support conclusions?

-Are there concerns about ethical or regulatory requirements being met?

Reviewer #1: The methods are clearly described. However, it is not clear, how the load of Histoplasma found in specific organs correlates to diagnostic accuracy. It depends on the clinical form of histoplasmosis. In pulmonary histoplasmosis, the lungs would probably have the highest loads, in disseminated forms it will depend on the organs which are infected. This can vary per patient. This is not addressed in the manuscript methods section.

line 163: this was postmortem material. Why could it have been limited? Why didn't the researchers take more material?

Reviewer #2: Objectives: the objective includes correlating fungal load, identifying optimal tissues, guiding clinical sampling, improving timeliness, and focusing on immunosuppressed individuals. This is overly broad. Please consider simplifying to Primary objective: Correlate fungal load with tissue distribution, and Secondary implication: Inform diagnostic strategies.

Study design is apropriated but lacks specificityis (see the attached document).

Population cleary described.

Sample size is sufficient.

Essential corrections are need on the statistical analysis (see the attached document).

Ethics approval is properly mentioned with the CAAE number.

**********

**Results**

-Does the analysis presented match the analysis plan?

-Are the results clearly and completely presented?

-Are the figures (Tables, Images) of sufficient quality for clarity?

Reviewer #1: The results are not well described. Especially the results concerning the answers to the research question are only presented in a table (table 1) and are not stratified by clinical form of histoplasmosis. THis should be presented much more elaborated and stratified by clinical disease form (disseminated histo, pulmonary histo, extrapulm histo) not only in one table but also in the text.

The main question of this study is to describe the added value of MITS/PCR, however the results do not show this added value.

Also, the added value of PCR in cases which were culture negative was not described. Were these true infections? Or were these only latent infections? And in these specific culture negative, PCR positive cases, which materials were PCR positive? What are the patient characteristics in these cases? Was histoplasmosis the cause of death or was there another cause of death and was Histoplasma found, but not of clinical relevance?

I miss the answers to the question: "How and when is MITS/PCR of added value?"

In table 2 I miss data on the diagnostics. Which tests of which organs were performed, and what were the results of culture and PCR of all materials of these patients. So add data from table 1 to table 2.

Lines 270-274: stratify these results according to clinical form (disseminated, lung. extrapulmonary). It makes no sense to present these data unstratified.

Reviewer #2: The data presented in the Results section are clinically important and potentially impactful, particularly given the high prevalence of histoplasmosis in advanced HIV. However the Results section requires statistical clarification, table restructuring, and removal of overinterpretation. Essential corrections include:

• Replace all “median ± SD” with appropriate statistical format.

• Clarify denominators for all percentages.

• Remove unsupported sensitivity/specificity claims.

• Define qPCR positivity threshold.

• Reformat Table 1 with a clear legend and standardized notation.

• Clearly explain classification criteria.

**********

**Conclusions**

-Are the conclusions supported by the data presented?

-Are the limitations of analysis clearly described?

-Do the authors discuss how these data can be helpful to advance our understanding of the topic under study?

-Is public health relevance addressed?

Reviewer #1: The conclusion is not supported by the data presented.

The limitations of the study are not clearly described.

In the discussion, several statements are made that are much broader than what can be concluded from the results of this study. These statements have not been investigated, and no sources are provided. As a result, it almost becomes a kind of 'expert opinion,' which is not the purpose of this study.

Line 331-332: we miss nuance on the meaning of a positive PCR. Is this 25,9% of the 18% with diagnosis histoplasmosis or 25,9% of the whole group. Please clarify, and also add this to the manuscript.

Line 361: rapid diagnosis: but then the clinicians should think about histoplasmosis first. Before you write, that this is a major problem in diagnostics.

Lines 363-368: the authors should be able to show resilts of this? They should have this information? I think this information is essential to describe, before you can conclude that PCR is superior to detect infections.

Line 380: this cannot be concluded. This is dependent of the localization of the infection and cannot be generalized like this.

Lines 395-397: this is not investigated in this study and no reference is added. So, please add a reference. Otherwise remove this section.

Lines 401-402: this is not investigated in this study and no reference is added. So, please add a reference. Otherwise remove this section.

Reviewer #2: All the discussion section must be worked. Please see the attached document for explanations.

**********

**Editorial and Data Presentation Modifications?**

Use this section for editorial suggestions as well as relatively minor modifications of existing data that would enhance clarity. If the only modifications needed are minor and/or editorial, you may wish to recommend “Minor Revision” or “Accept”.

Reviewer #1: Minor comments: Throughout the manuscript 0, 1 or 2 decimals are used in the Ct values. Please use 1 decimal for Ct values throughout the manuscript.

line 105-107: grammar not OK, please rephrase

line 137: change 2,063,547 into approximately 2 million

Line 156: change colected into collected

Table 1: Wat does LCR mean?

Table 1: what do the numbers 43, 44, 28, 22 and so on mean? I suggest Ct values. Please clarrify and add to the text.

Line 271: what do the authors mean with 81TT.4%?

Line 311: change people living with HIV into PLHIV

Line 331: change them into they

Line 349: write Histoplasma in italics

Reviewer #2: The manuscript needs major revision. See suggestions ont the attached document.

**********

**Summary and General Comments**

Use this section to provide overall comments, discuss strengths/weaknesses of the study, novelty, significance, general execution and scholarship. You may also include additional comments for the author, including concerns about dual publication, research ethics, or publication ethics. If requesting major revision, please articulate the new experiments that are needed.

Reviewer #1: The study describes an interesting technique for the diagnosis of histoplasmosis in HIV postive patients. The methods are clearly described. However the results are not well described and do not support the conclusions made. The conclusions in the discussion cannot be drawn from this study.

Abstract: Conclusion too strong

Author summary: Conclusion too strong and specify what is the gold standaard?

Reviewer #2: The manuscript requires major revision, as several points need clarification and further development. We consider high scientific relevance, and high impact potential after revision. However, the follow score was given to:

• Methodological robustness: ★★★★☆

• Interpretative caution: ★★★☆☆

• Clarity and conciseness: ★★★☆☆

**********

PLOS authors have the option to publish the peer review history of their article (what does this mean?). If published, this will include your full peer review and any attached files.

If you choose “no”, your identity will remain anonymous but your review may still be made public.

**Do you want your identity to be public for this peer review?**  For information about this choice, including consent withdrawal, please see our Privacy Policy.

Reviewer #1: No

Reviewer #2: No

[NOTE: If reviewer comments were submitted as an attachment file, they will be attached to this email and accessible via the submission site. Please log into your account, locate the manuscript record, and check for the action link "View Attachments". If this link does not appear, there are no attachment files.]

**Figure resubmission:**

While revising your submission, we strongly recommend that you use PLOS’s NAAS tool (https://ngplosjournals.pagemajik.ai/artanalysis) to test your figure files. NAAS can convert your figure files to the TIFF file type and meet basic requirements (such as print size, resolution), or provide you with a report on issues that do not meet our requirements and that NAAS cannot fix.

After uploading your figures to PLOS’s NAAS tool - https://ngplosjournals.pagemajik.ai/artanalysis, NAAS will process the files provided and display the results in the "Uploaded Files" section of the page as the processing is complete. If the uploaded figures meet our requirements (or NAAS is able to fix the files to meet our requirements), the figure will be marked as "fixed" above. If NAAS is unable to fix the files, a red "failed" label will appear above. When NAAS has confirmed that the figure files meet our requirements, please download the file via the download option, and include these NAAS processed figure files when submitting your revised manuscript.

**Reproducibility:**

To enhance the reproducibility of your results, we recommend that authors of applicable studies deposit laboratory protocols in protocols.io, where a protocol can be assigned its own identifier (DOI) such that it can be cited independently in the future. Additionally, PLOS ONE offers an option to publish peer-reviewed clinical study protocols. Read more information on sharing protocols at https://plos.org/protocols?utm_medium=editorial-email&utm_source=authorletters&utm_campaign=protocols

Attachment Submitted filename: Review_ manuscript PNTD-D-25-01973.pdf

# Author comment: Author response to Decision Letter 1

**DOI:** 10.1371/journal.pntd.0014545.r002

17 Jun 2026

Attachment Submitted filename: response.docx

# Peer review: Decision Letter 1

**DOI:** 10.1371/journal.pntd.0014545.r003

**Authors:** Marcio Rodrigues

3 Jul 2026

Dear dr Baia,

We are pleased to inform you that your manuscript 'Tissue distribution of Histoplasma capsulatum in deceased people living with HIV assessed by minimally invasive tissue sampling: an autopsy-based case series in the Brazilian Amazon' has been provisionally accepted for publication in PLOS Neglected Tropical Diseases.

Before your manuscript can be formally accepted you will need to complete some formatting changes, which you will receive in a follow up email. A member of our team will be in touch with a set of requests.

Please note that your manuscript will not be scheduled for publication until you have made the required changes, so a swift response is appreciated.

IMPORTANT: The editorial review process is now complete. PLOS will only permit corrections to spelling, formatting or significant scientific errors from this point onwards. Requests for major changes, or any which affect the scientific understanding of your work, will cause delays to the publication date of your manuscript.

Should you, your institution's press office or the journal office choose to press release your paper, you will automatically be opted out of early publication. We ask that you notify us now if you or your institution is planning to press release the article. All press must be co-ordinated with PLOS.

Thank you again for supporting Open Access publishing; we are looking forward to publishing your work in PLOS Neglected Tropical Diseases.

Best regards,

Marcio L Rodrigues

Section Editor

PLOS Neglected Tropical Diseases

Marcio Rodrigues

Section Editor

PLOS Neglected Tropical Diseases

Shaden Kamhawi

co-Editor-in-Chief

PLOS Neglected Tropical Diseases

orcid.org/0000-0003-4304-636XX

Paul Brindley

co-Editor-in-Chief

PLOS Neglected Tropical Diseases

orcid.org/0000-0003-1765-0002

***********************************************************

p.p1 {margin: 0.0px 0.0px 0.0px 0.0px; line-height: 16.0px; font: 14.0px Arial; color: #323333; -webkit-text-stroke: #323333}span.s1 {font-kerning: none

Reviewer's Responses to Questions

**Key Review Criteria Required for Acceptance?**

As you describe the new analyses required for acceptance, please consider the following:

**Methods**

-Are the objectives of the study clearly articulated with a clear testable hypothesis stated?

-Is the study design appropriate to address the stated objectives?

-Is the population clearly described and appropriate for the hypothesis being tested?

-Is the sample size sufficient to ensure adequate power to address the hypothesis being tested?

-Were correct statistical analysis used to support conclusions?

-Are there concerns about ethical or regulatory requirements being met?

Reviewer #2: Following a careful revision of the original submitted manuscript by the authors and in response to the reviewers' comments, we consider the manuscript suitable for publication in PLOS Neglected Tropical Diseases. The study objectives are clearly articulated, and a clear, testable hypothesis is stated. The study design is appropriate to address the objectives, and the study population is well described and appropriate for testing the proposed hypothesis. The sample size was sufficient to provide adequate statistical power to test the study hypothesis. The statistical analyses were appropriate and correctly applied, and there are no concerns regarding ethical or regulatory requirements.

**********

**Results**

-Does the analysis presented match the analysis plan?

-Are the results clearly and completely presented?

-Are the figures (Tables, Images) of sufficient quality for clarity?

Reviewer #2: Regarding the Results, they are clearly and comprehensively presented. The figures, tables, and images are clear, of high quality, and effectively support the findings.

**********

**Conclusions**

-Are the conclusions supported by the data presented?

-Are the limitations of analysis clearly described?

-Do the authors discuss how these data can be helpful to advance our understanding of the topic under study?

-Is public health relevance addressed?

Reviewer #2: The data presented strongly support the conclusions, and the study limitations are clearly described. The authors discuss how their findings contribute to a better understanding of the histopathology of histoplasmosis. The manuscript is of significant public health relevance.

**********

**Editorial and Data Presentation Modifications?**

Use this section for editorial suggestions as well as relatively minor modifications of existing data that would enhance clarity. If the only modifications needed are minor and/or editorial, you may wish to recommend “Minor Revision” or “Accept”.

Reviewer #2: No modifications.

**********

**Summary and General Comments**

Use this section to provide overall comments, discuss strengths/weaknesses of the study, novelty, significance, general execution and scholarship. You may also include additional comments for the author, including concerns about dual publication, research ethics, or publication ethics. If requesting major revision, please articulate the new experiments that are needed.

Reviewer #2: The revised manuscript has been substantially improved in response to the reviewers' comments and is now suitable for publication in PLOS Neglected Tropical Diseases. The study addresses an important topic with clear public health relevance and provides valuable insights into the histopathology of histoplasmosis.

The research objectives are well defined, and the study is guided by a clear, testable hypothesis. The study design, population, sample size, and statistical analyses are appropriate and adequately support the study objectives. Ethical and regulatory aspects have been appropriately addressed.

The results are clearly and comprehensively presented, and the figures, tables, and images are of high quality and effectively illustrate the findings. The conclusions are well supported by the data, and the study limitations are appropriately acknowledged and discussed. Overall, the manuscript makes a meaningful contribution to the understanding of the histopathology of histoplasmosis and will be of interest to both researchers and clinicians in the field of neglected tropical diseases.

**********

PLOS authors have the option to publish the peer review history of their article (what does this mean?). If published, this will include your full peer review and any attached files.

If you choose “no”, your identity will remain anonymous but your review may still be made public.

**Do you want your identity to be public for this peer review?**  For information about this choice, including consent withdrawal, please see our Privacy Policy.

Reviewer #2: No

# Editor report: Acceptance letter

**DOI:** 10.1371/journal.pntd.0014545.r004

**Authors:** Marcio Rodrigues

Dear dr Baia,

We are delighted to inform you that your manuscript, "Tissue distribution of Histoplasma capsulatum in deceased people living with HIV assessed by minimally invasive tissue sampling: an autopsy-based case series in the Brazilian Amazon," has been formally accepted for publication in PLOS Neglected Tropical Diseases.

We have now passed your article onto the PLOS Production Department who will complete the rest of the publication process. All authors will receive a confirmation email upon publication.

The corresponding author will soon be receiving a typeset proof for review, to ensure errors have not been introduced during production. Please review the PDF proof of your manuscript carefully, as this is the last chance to correct any scientific or type-setting errors. Please note that major changes, or those which affect the scientific understanding of the work, will likely cause delays to the publication date of your manuscript. Note: Proofs for Front Matter articles (Editorial, Viewpoint, Symposium, Review, etc...) are generated on a different schedule and may not be made available as quickly.

Soon after your final files are uploaded, the early version of your manuscript will be published online unless you opted out of this process. The date of the early version will be your article's publication date. The final article will be published to the same URL, and all versions of the paper will be accessible to readers.

For Research Articles, you will receive an invoice from PLOS for your publication fee after your manuscript has reached the completed accept phase. If you receive an email requesting payment before acceptance or for any other service, this may be a phishing scheme. Learn how to identify phishing emails and protect your accounts at https://explore.plos.org/phishing.

Thank you again for supporting open-access publishing; we are looking forward to publishing your work in PLOS Neglected Tropical Diseases.

Best regards,

Shaden Kamhawi

co-Editor-in-Chief

PLOS Neglected Tropical Diseases

Paul Brindley

co-Editor-in-Chief

PLOS Neglected Tropical Diseases
